# SARS-CoV-2 seroprevalence in Mongolia: Results from a national population survey

**DOI:** 10.1016/j.lanwpc.2021.100317

**Published:** 2021-11-23

**Authors:** Battogtokh Chimeddorj, Undram Mandakh, Linh-Vi Le, Batzorig Bayartsogt, Zolzaya Deleg, Oyunsuren Enebish, Oyunbaatar Altanbayar, Battur Magvan, Anuujin Gantumur, Otgonjargal Byambaa, Gerelmaa Enebish, Bat-Erdene Saindoo, Mandakhnaran Davaadorj, Avarzed Amgalanbaatar, Khangai Enkhtugs, Usukhbayar Munkhbayar, Batkhuu Bayanjargal, Tuyajargal Badamsambuu, Myagmartseren Dashtseren, Zolmunkh Narmandakh, Khongorzul Togoo, Enkh-Amar Boldbaatar, Ariunzaya Bat-Erdene, Yerkyebulan Mukhtar, Oyu-Erdene Shagdarsuren, Mandukhai Ganbat, Ochbadrakh Batjargal, Bayasgalantai Bavuusuren, Batzaya Batchuluun, Gereltsetseg Zulmunkh, Ganbaatar Byambatsogt, Khurelbaatar Nyamdavaa, Tserendagva Dalkh, Damdindorj Boldbaatar, Tuvshinjargal Tseren, Darambazar Gantulga, Otgonbayar Damdinbazar, Byambasuren Vanchin, Lorenzo Subissi, Isabel Bergeri, Davaalkham Dambadarjaa, Nymadawa Pagbajabyn, Gregory Greif, Ryenchindorj Erkhembayar

**Affiliations:** a Department of Microbiology and Infection Prevention Control, School of Biomedicine, Mongolian National University of Medical Sciences, Ulaanbaatar, Mongolia; b Institute of Biomedical Sciences, Mongolian National University of Medical Sciences, Ulaanbaatar, Mongolia; c Mongolia Japan Hospital, Mongolian National University of Medical Sciences, Ulaanbaatar, Mongolia; d Department of Family Medicine, School of Medicine, Mongolian National University of Medical Sciences, Ulaanbaatar, Mongolia; e World Health Organization, Regional Office for the Western Pacific, Manila, Philippines; f Department of Epidemiology and Biostatistics, School of Public Health, Mongolian National University of Medical Sciences, Ulaanbaatar, Mongolia; g Department of Planning and Research, Ministry of Health, Ulaanbaatar, Mongolia; h Department of International Cyber Education, Graduate School, Mongolian National University of Medical Sciences, Ulaanbaatar, Mongolia; i Department of Immunology, School of Biomedicine, Mongolian National University of Medical Sciences, Ulaanbaatar, Mongolia; j Department of Biochemistry, School of Biomedicine, Mongolian National University of Medical Sciences, Ulaanbaatar, Mongolia; k Mongolian National University of Medical Sciences, Ulaanbaatar, Mongolia; l Department of Cardiology, School of Medicine, Mongolian National University of Medical Sciences, Ulaanbaatar, Mongolia; m World Health Organization, Geneva, Switzerland; n Mongolian Academy of Sciences, Ulaanbaatar, Mongolia

**Keywords:** SARS-CoV-2, COVID-19, Pandemics, Seroepidemiological studies, Mongolia, Seroprevalence, Immunity

## Abstract

**Background:**

With the global spread of severe acute respiratory syndrome coronavirus 2 (SARS-CoV-2) in early 2020, Mongolia implemented rapid emergency measures and did not report local transmission until November 2020. We conducted a national seroprevalence survey to monitor the burden of SARS-CoV-2 in Mongolia in the months surrounding the first local transmission.

**Methods:**

During October-December 2020, participants were randomly selected using age stratification and invited for interviews and blood samples at local primary health centres. We screened for total SARS-CoV-2 antibodies, followed by two-step quantitative SARS-CoV-2 IgG serology tests for positive samples. Weighted and test-adjusted seroprevalences were estimated. We used chi-square, Fisher's exact and other tests to identify variables associated with seropositivity.

**Findings:**

A total of 5000 subjects were enrolled. We detected SARS-CoV-2 IgG antibodies in 72 samples. Crude seroprevalence of SARS-CoV-2 antibodies was 1·44% (95%CI,1·21-1·67). Population weighted and test-adjusted seroprevalences were 1·36% (95%CI,1·11-1·63) and 1·45% (95%CI,1·11-1·63), respectively. Age, sex, geographical, and occupational factors were not associated with seropositivity (p>0·05). Symptoms and signs within past 3 months and seropositivity were not associated at the time of the survey (p>0·05).

**Interpretation:**

SARS-CoV-2 seroprevalence in Mongolia was low in the first year of the pandemic potentially due to strong public health measures, including border restrictions, educational facilities closure, earlier adoption of mask-wearing and others. Our findings suggest large-scale community transmission could not have occurred up to November 2020 in Mongolia. Additional serosurveys are needed to monitor the local pandemic dynamic and estimate how far from herd immunity Mongolia will be following-up with vaccination programme in 2021 and 2022.

**Funding:**

World Health Organisation, WHO UNITY Studies initiative, with funding by the COVID-19 Solidarity Response Fund and the German Federal Ministry of Health (BMG) COVID-19 Research and development.

**Translation:**

Cyrillic and Traditional Mongolian translation of abstract is available on appendix section.

**Research in Context**

**Evidence before this study**

We systematically searched PubMed and the “SeroTracker” dashboard, a collaborative engine for seroprevalence studies, for SARS-CoV-2 seroepidemiological studies around the world. We found 704 results using MeSH terms Seroepidemiological Studies AND (SARS-CoV-2 OR COVID-19) on PubMed as of September 20, 2021. We added “AND Asia” to the search and found 116 entries. Only one investigation relevant to MERS in wildlife was found for Mongolia.

According to the SeroTracker dashboard, 2965 surveys covering 23·5 million participants over 116 countries were registered as of September 20, 2021. No entry was seen from Mongolia at a local, regional, or countrywide level.

The Mongolian Ministry of Health has had a national SARS-CoV-2 notification system since early 2020, and RT-PCR based testing confirmed cases on a daily basis. Daily confirmed cases and nationwide status are available in the appendix section (appendix 3).

**Added value of this study**

This was the first nationwide investigation for SARS-CoV-2 seroepidemiological study from Mongolia. Data collection in our study covers early onset of community transmission in late 2020, capturing transmission over the first year of the pandemic when the true extent of transmission was unknown, particularly for remote areas where testing and reporting capacity were low. We were able to establish that despite low case notifications, and zero notifications in some provinces, there was transmission across the country by the end of 2020, albeit at low levels. The general public of Mongolia was minimally exposed for the first year of the global pandemic, before vaccines became available.

We document another well-contained practice for SARS-CoV-2 infection from the Western Pacific region that had the lowest seroprevalence by end 2020.

**Implications of all the available evidence**

Public health measures involving border closures, quarantine and isolation, and strict movement restrictions, business and school closures, taken rapidly nationwide during the early stage of the pandemic can be beneficial particularly in lower-and-middle income countries with weaker health systems. This allows countries to plan response measures, gather evidence and prepare for combatting the larger outbreaks in resource limited settings. Ultimately, these public health measures prevented surges in SARS-CoV-2 cases as observed in many countries in the first year of the COVID-19 pandemic and before vaccines become available in Mongolia.

Alt-text: Unlabelled box

## Introduction

COVID-19 was first reported in Wuhan Province of the People's Republic of China (PRC) on December 31, 2019.^1^ By the end of 2020, the reported seroprevalence of SARS-CoV-2 ranged globally from 1·7% in the Western Pacific to 19·6% in Southeast Asia.^1^ Countries with weaker containment measures had relatively high seroprevalence in early 2020, and some countries even reported potential herd immunity among the general population by natural infection before vaccinations became available.^3^^,^^4^ On the other hand, strong preventive measures and fast action by some governments resulted in relatively swift containment of the virus.

Mongolia is a lower-middle income, landlocked country situated between the Russian Federation and PRC with a population of 3·3 million. In January 2020 the Mongolian government responded with rapid measures to contain SARS-CoV-2.^1^ The early response and preventive measures were exemplary among low- and middle-income countries (LMICs): mandatory mask-wearing was enforced early, and after closing the border on March 10, 2020, all incoming travellers went into a mandatory facility quarantine. Following border closure, other restrictions such as school closures were implemented on a rolling basis.^1^ An RT-PCR testing-based sentinel surveillance system was established by mid-January 2020 to monitor local outbreaks across the country, with sampling taken from both random samples and subjects presenting symptoms.^1^ It covered an average of 288 tests per 10,000 population and presented a 0·5% positivity rate up to mid-November, when local cases have not yet been reported.^1^ Mongolia did not report excess mortality in 2020, despite highly industrialised nations such as the USA and UK reporting near 50 and 100 percent excess mortality rates.^7^^,^^8^ The first local transmission was detected in November of 2020 through transmission from an index case arriving into Mongolia via the Russian border.^9^^,^^10^

A nationally representative serosurvey was essential for the most dispersedly populated country within the Western Pacific region to supplement the epidemiological data from RT-PCR monitoring, and to determine true extent of transmission in Mongolia. In this study, we aimed to measure the seroprevalence of SARS-CoV-2 in Mongolia at the end of 2020, one year into the global COVID-19 pandemic, with the goal of estimating the extent to which the population had been exposed to the virus and to identify factors associated with seropositivity before the vaccine rollout that began on February 23, 2021.

## Methods

### Study design and study population

We conducted a national population-based seroepidemiological survey in Mongolia for SARS-CoV-2 antibodies during October-December 2020 using multistage, age-stratified cluster sampling. Clusters of 100 subjects were gathered in proportion to age strata and the size of the urban (Ulaanbaatar city) and rural populations. Mongolia is administratively divided into 21 provinces and Ulaanbaatar city, forming 5 economic regions: Western, Khangai, Central, Ulaanbaatar, and Eastern. The following 9 provinces were randomly selected such that at least 2 provinces (three for central) represented each of the 4 rural regions: Bayan-Ulgii, Zavkhan, Bayankhongor, Orkhon, Selenge, Umnugovi, Dornogovi, Khentii and Dornod. In each province, three cluster sites including the provincial centre and two other soums (smaller administrative regions within the province) were selected. For Ulaanbaatar, where over one-half of Mongolia's population resides, twenty-three khoroos (smaller administrative regions within the capital) from six central districts were randomly selected. The detailed sampling strategy and population size used for the study are available in Appendix 2. All 50 clusters ensured nationally representative sampling, also available in Appendix 2.

### Study protocol and Sample clusters

At each cluster site subject selection was based upon age strata and matching current address to the site. Participants in the following groups were considered ineligible for enrolment: those who expressed voluntary refusal; those contraindicated for venepuncture; and returnees from international travel or known imported cases of SARS-CoV-2. For this study we adapted the WHO UNITY Studies for population-based, age-stratified seroepidemiological investigation protocol.^1^ The protocol is registered to a WHO compliant system under registry #2020·9·MOG·1·ESR, October 22, 2020. We obtained ethical clearance from the research ethical review boards at the Ministry of Health, Mongolia (Resolution #193, October 1^st^, 2020) and World Health Organization Regional Office for the Western Pacific.

### Study timeline

Mongolia reported the first imported case on March 10, 2020 and did not report any domestic cases until November 10, 2020. We collected the data between October 13 and December 4 in 2020. All rural samples were collected before the notification of the first RT-PCR confirmed locally transmitted case. Urban sample collections were conducted from mid-November to December 4, 2020. A timeline showing RT-PCR confirmed cases and survey implementation is illustrated in Appendix 3.

### Procedures

We used the simple random selection process to select participants from national civil registration through local primary health centres. Consent forms were provided and signed for participation and blood sampling, and parental consent was taken for children. The research team filled out the survey questionnaire through interviews and collected 5 ml and 3 ml of venous blood into gel separation tubes from adults and children, respectively. Serum extracts were aliquoted into 2 or 3, and stored for laboratory testing. All serum samples were transported in portable refrigerators at -20 ºC, stored in -80ºC deep freezers and later analysed at the laboratory of Mongolia Japan Teaching Hospital, Mongolian National University of Medical Sciences in Ulaanbaatar, Mongolia.

All serum samples were screened for total antibodies to SARS-CoV-2 using the Wantai, SARS-CoV-2 Ab ELISA, WS-1096 (Beijing Wantai Biological Pharmacy Enterprise) product manual. This test was chosen because of its high sensitivity and specificity, as assessed in multiple independent evaluation studies.^1^ Positive and borderline reactive samples were repeatedly tested by Wantai. Subsequently, positive samples were processed by two-step, confirmatory quantitative SARS-CoV-2 IgG assay (Kantaro, Quantitative SARS-CoV-2 IgG Antibody RUO Kit for detecting antibodies to receptor binding domain [RBD] and spike protein [SP] antigens). In addition, samples with discordant results for total antibodies were tested by confirmatory SARS-CoV-2 IgG (anti-RBD and anti-SP) testing to maximise case detection. A case was positive for SARS-CoV-2 antibodies if (i) positive for total antibodies to SARS-CoV-2 and two-step IgG assay or (ii) negative to total antibodies and positive for SARS-CoV-2 IgG (anti-RBD and anti-SP) with detectable anti-SP IgG titre. This laboratory algorithm, which included testing with a second serology assay, was used to minimise the high false positivity rate expected in low prevalence settings. Both assays were read by a RT-6900, Rayto microplate reader. We analysed the IgG antibody level (arbitrary units per millilitre, AU/mL) of the anti-SP for positive samples.

### Data collection

Study teams collected information on demographic characteristics including residential address, ethnicity, and occupation type (health or non-health). We included three variables for COVID-19 preventive behaviour, four variables for contact exposure, 20 variables for acute and general clinical symptoms, four variables for additional symptoms, three variables for hospitalisation or school/work absence due to symptoms, and lastly, 15 variables for past medical history and pregnancy. A total of 55 independent variables were available for analysis. Participants were asked to recall symptoms and COVID-19 related items from the previous 3 months.

### Statistical analysis

We estimated a sample size of 4750 subjects based on an expected 1% prevalence of SARS-CoV-2 antibodies a 0·4% margin of error, a design effect of 2 and total population of 3,238,479 inhabitants (2018).^1^ We increased the sample size by 5% to account for refusals and missing data and rounded up to obtain a target sample size of 5000 subjects for even clusters of 100 participants.

We computed the seroprevalence of SARS-CoV-2 as the percentage of SARS-CoV-2 antibodies positive in the total study population, with 95 confidence intervals. We used a bootstrapping method with 10,000 samples to compute confidence intervals for seroprevalence ratios. We estimated weighted prevalence rates according to age, sex, and provincial or district population size in Mongolia using 2020 population data.^1^ Stratification factors including geographical location, sex and age were excluded from weighted prevalence estimations for the stratum. We adjusted the seroprevalence for test sensitivity of primary total antibodies test (Wantai). Weighted and test adjusted estimates made independently. Full methods for weighted and adjusted estimates and additional mapping are available in Appendix 4. Chi-square and Fisher's exact tests were used to identify associations of SARS-CoV-2 seropositivity with clinical symptoms (odd's ratio), risk factors and other variables (prevalence ratio). ‘Do not know’ answers or missing variables were excluded from inferential analysis for associations of seropositivity with clinical symptoms or other factors. Additionally, we explored with multivariate logistic regression analysis to further identify factors associated to seropositivity, adjusting for age group and sex as covariates. We used independent samples T-test for SARS-CoV-2 IgG titre analysis expressed in mean±SEM, after checking for the normality of distribution. Inferential analysis was based on the crude seroprevalences.

### Role of funding source

The sponsor of the study had no direct role in the study design, data collection, data analysis, data interpretation, or writing of the report. The corresponding and lead authors had full access to all data.

## Results

**Figure 1:**
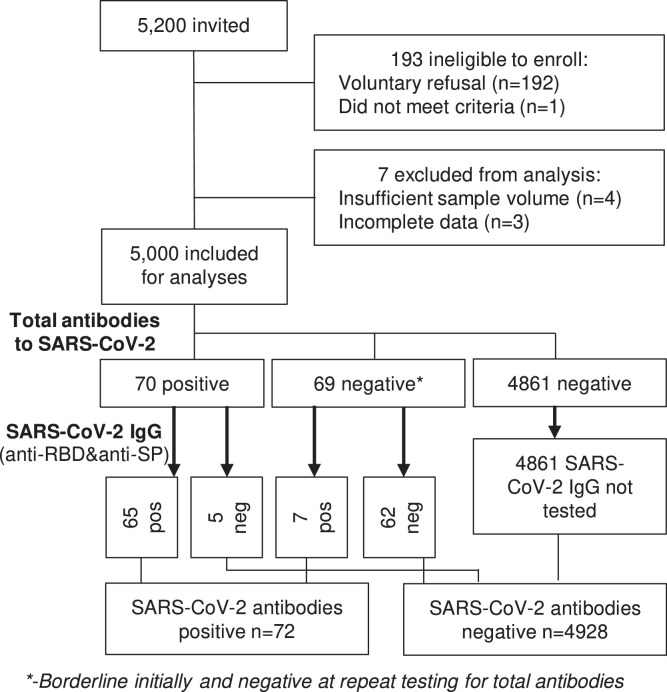
Flowchart and study profile.

From 5200 individuals invited to the study, 5000 (96·2%) were included (Figure 1). Females represented 64·8% (n=3242) of the participants, and the participant selection matched to age group stratums proportionate to Mongolian population size. Among the study subjects, 1898 (38·0%) were children or adolescents under 20 years. We recruited 816 subjects aged 20 to 29 (16·3%) years and 823 aged (16·5%) 30 to 39 years. A total of 2300 (46·0%) subjects were recruited in urban settings. We included 4073 (81·5%) Khalkha ethnicity, the predominant Mongolian subgroup, and 315 (6·3%) Kazakh minorities. A total of 588 (11·8%) participants had an occupation associated with healthcare facilities, Table 1.

**Table 1:** Participants’ demographic and general characteristics of SARS-CoV-2 serosurvey in Mongolia, October-December, 2020.

		n	%
Total			
		5000	100
**Residential location**			
	Rural	2700	54·00
	Urban	2300	46·00
**Sex**			
	Female	3242	64·84
	Male	1758	35·16
**Age in years**			
	0 to 4	579	11·58
	5 to 9	559	11·18
	10 to 14	430	8·60
	15 to 19	330	6·60
	20 to 29	816	16·32
	30 to 39	823	16·46
	40 to 49	677	13·54
	50 to 59	468	9·36
	60 to 69	218	4·36
	70 and above	99	1·98
	Missing	1	0·02
**Ethnicity**			
	Khalkha	4073	81·46
	Kazakh	315	6·30
	Buryat	214	4·28
	Others	171	3·42
	Durvud	55	1·10
	Missing	172	3·44
**Occupational type**			
	Non-health sector	3456	69·12
	Healthcare worker	588	11·76
	Missing	956	19·12
**Comorbidity (1 or more)**			
	No	4784	95·68
	Yes	216	4·32
**Contact with COVID-19 confirmed case**			
	No	4904	98·08
	Yes	69	1·38
	Unknown	26	0·52
	Missing	1	0·02

Among the 5000 serum samples analysed, 70 were positive for total antibodies for SARS-CoV-2. Of these, 65 cases were confirmed by the two-step quantitative IgG assay and five were negative. In addition, seven SARS-CoV-2 IgG positive cases were identified by confirmatory tests among 69 negatives samples from the initial total antibodies test (borderline and determined negative for retest at Wantai). A total of 4928 samples tested negative (Figure 1). The seroprevalence of SARS-CoV-2 was 1·44% (n=72/5000; 95%CI, 1·21-1·67) (Table 2). The seven cases who were negative on the total antibodies to SARS-CoV-2 test had slightly lower anti-spike protein IgG titre than positive samples from the initial test. However, this difference was not significant: 7·99±2·20 and 13·08±3·25 AU/ml respectively.

**Table 2:** Prevalence of SARS-CoV-2 antibodies in Mongolia by region, sex and age in late 2020.

	N, total	n, seropositive	Crude prevalence rate	Weighted prevalence rate*	Test adjusted prevalence rate **
**Total**	5000	72	1·44 (1·21-1·67)	1·36 (1·11-1·63)	1·45 (1·11-1·63)
**Residential location**					
Urban	2300	30	1·3 (0·99-1·64)	1·31 (0·98-1·67)	1·39 (0·98-1·67)
Rural	2700	42	1·55 (1·24-1·89)	1·44 (1·13-1·76)	1·53 (1·13-1·76)
**Sex**					
Female	3242	51	1·57 (1·27-1·88)	1·45 (1·15-1·77)	1·54 (1·15-1·77)
Male	1758	21	1·19 (0·85-1·56)	1·28 (0·89-1·7)	1·35 (0·89-1·7)
**Age in years**					
0 to 4	579	8	1·38 (0·76-2·08)	1·85 (1·0-2·88)	1·97 (1·0-2·88)
5 to 9	559	1	0·18 (0·0-0·45)	0·1 (0·0-0·26)	0·1 (0·0-0·26)
10 to 14	430	6	1·4 (0·69-2·2)	1·96 (0·87-3·28)	2·09 (0·87-3·28)
15 to 19	330	5	1·52 (0·63-2·52)	1·24 (0·52-2·1)	1·32 (0·52-2·1)
20 to 29	816	15	1·83 (1·21-2·53)	1·51 (0·94-2·16)	1·6 (0·94-2·16)
30 to 39	823	19	2·31 (1·6-3·07)	1·98 (1·29-2·74)	2·11 (1·29-2·74)
40 to 49	677	11	1·63 (1·0-2·33)	1·45 (0·79-2·25)	1·54 (0·79-2·25)
50 to 59	468	3	0·63 (0·21-1·19)	0·38 (0·11-0·72)	0·4 (0·11-0·72)
60 to 69	218	3	1·37 (0·43-2·58)	1·81 (0·43-3·57)	1·92 (0·43-3·57)
70 and above	99	1	1·0 (0·0-2·63)	0·62 (0·0-1·6)	0·65 (0·0-1·6)
**Occupational type**					
Non-health sector	3456	46	1·33 (1·07-1·6)	1·19 (0·92-1·48)	1·26 (0·92-1·48)
Healthcare worker	588	10	1·7 (1·01-2·49)	1·58 (0·89-2·35)	1·68 (0·89-2·35)
**Comorbidity (1 or more)**					
Yes	216	3	1·39 (0·45-2·59)	1·09 (0·28-2·15)	1·15 (0·28-2·15)
No	4784	69	1·44 (1·2-1·68)	1·37 (1·11-1·65)	1·46 (1·11-1·65)

Rate% (95%CI, Lower and Upper Bound), *- Stratification factor not included for weighted estimation, **-Adjusted rate for Wantai, total antibodies test sensitivity

**Figure 2:**
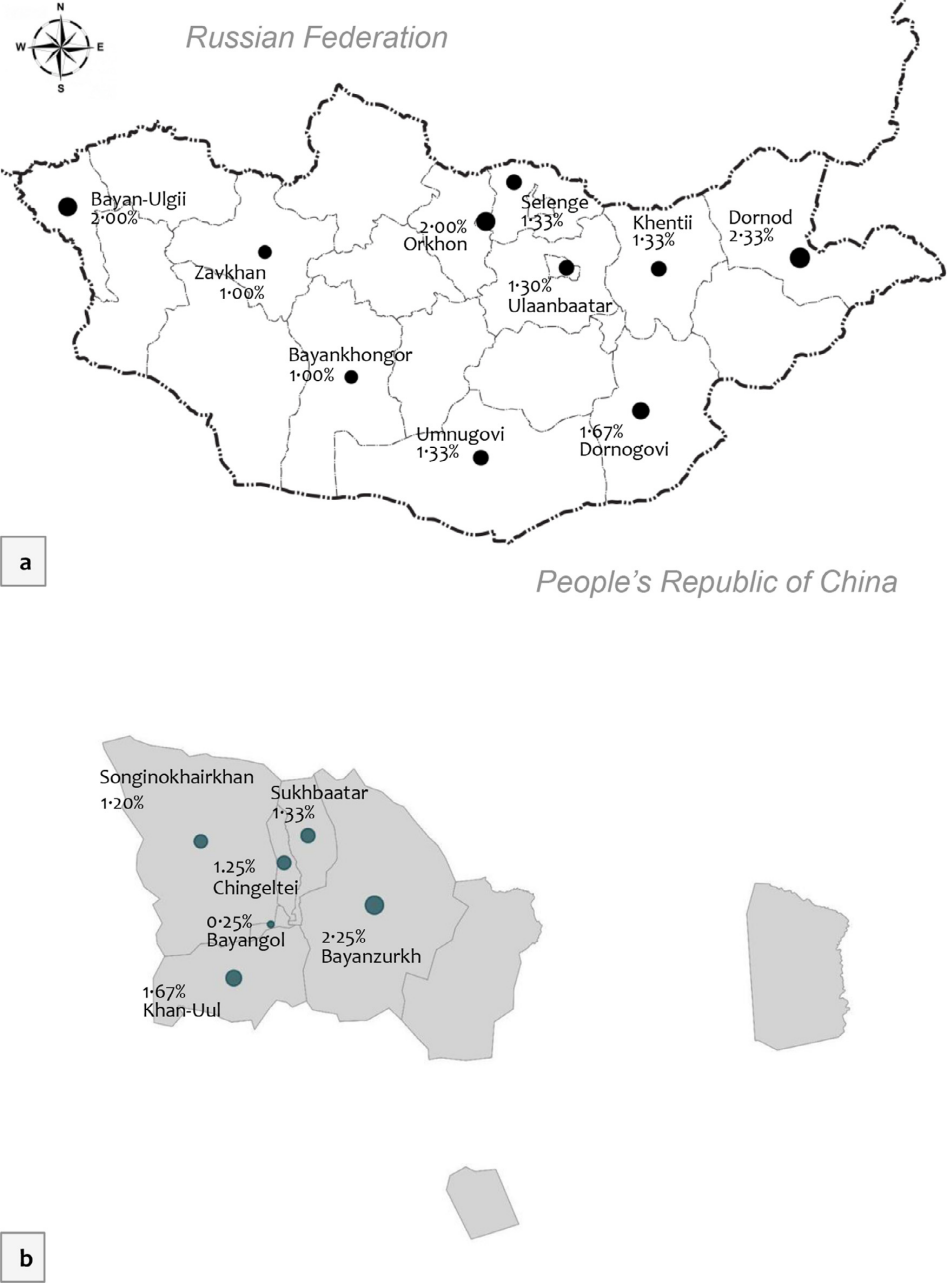
Geographical distribution of SARS-CoV-2 seroprevalence (crude) in Mongolia; a. Provinces, rural parts, b. Districts of Ulaanbaatar, urban population, late 2020.

The crude seroprevalence of SARS-CoV-2 was similar between urban and rural population, sex, age groups, occupation type, and ethnicity subgroups (Table 2). Figure 2 illustrates the geographical distribution of SARS-CoV-2 in Mongolia. Among provinces the seroprevalence ranged between 1·00% and 2·33%, with an average of 1·55% (95%CI, 1·24-1·89) for the rural population. Dornod province had the highest seroprevalence at 2·3% (95%CI, 1·2-3.6), followed by Bayan-Ulgii and Orkhon, each with 2·0% (6/300; 95%CI, 1·0-3.2). There was no statistically significant difference in seroprevalence across the rural provinces (p>0.05). the seroprevalence for districts in Ulaanbaatar ranged between 0.25% to 2.25%, figure 2, caption b. Bayanzurkh district had a crude seroprevalence of 2·25% (9/400; 95%CI, 1·3-3.35), whilst Bayangol district had a seroprevalence of 0·25% (1/400; 95%CI, 0·12-0.65). The crude seroprevalence was 1·30% (95%CI, 0·99-1·64) in urban areas, and the 6 districts of Ulaanbaatar had no statistically significant differences (p>0·05). Detailed soum and khoroo level prevalence maps are available in Appendix 4. As of early December 2020, at least 48,349 (95%CI; 40,626 – 56,071) individuals could have been exposed to SARS-CoV-2 according to the crude seroprevalence for the 2020 population size.

The weighted seroprevalence was 1·36% for SARS-CoV-2 antibodies (Table 2). Rural and urban population had minimally lower seroprevalences after weighted adjustment, with 1·31% (0·98-1·67) and 1·44% (1·13-1·76) respectively. After adjusting for test sensitivity, the seroprevalence of SARS-CoV-2 was marginally higher in the rural population than in the urban population (Table 2).

The seroprevalence was 1·57% (51/3242; 95%CI, 1·27-1·88) for females and 1·19% (21/1758; 95%CI, 0·85-1·56) for males. For age groups, young adults aged between 30 to 39 had a 2·31% (19/823; 95%CI, 1·6-3·07) seroprevalence rate, followed by adults aged 20 to 29 with a rate of 1·83% (15/816; 95%CI, 1·21-2·53). Five to 9-year-old children had a seroprevalence of 0·18% (1/559; 95%CI, 0·0-0·45). Older adults of 50 to 59 years old had a seroprevalence of 0·63% (3/468; 95%CI, 0·21-1·19). Between the age groups there were no significant differences for SARS-CoV-2 seroprevalence (p=0·123). After weighted adjustment estimates, children aged 0-4; 10-14 and senior adults (60-69) had slightly higher prevalence than the crude seroprevalence by 0·47, 0·56, and 0·44 percent respectively, Table 2. Other age groups, males and females, and rural and urban population had slightly lower prevalence according to the weighted prevalence, p>0·05.

**Figure 3:**
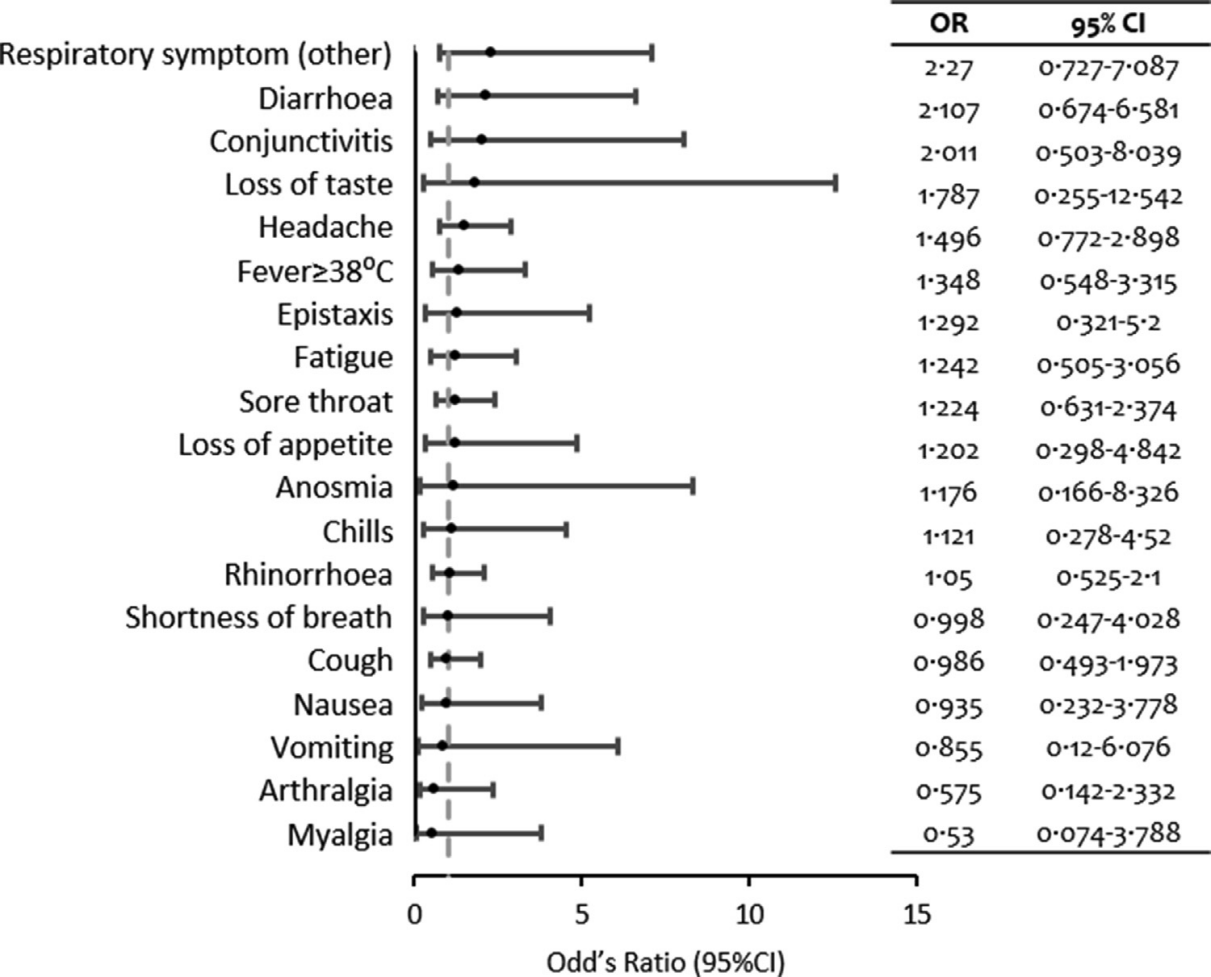
Clinical findings and seropositivity of SARS-CoV-2 in late 2020.

Among the symptoms and signs collected, respiratory symptoms other than acute lower and upper tract infection symptoms were reported twice as often among the seropositive participants (OR=2·27; 95%CI 0·73-7·09) compared to seronegative subjects. Seropositive subjects also experienced symptoms and signs including diarrhoea, conjunctivitis, ageusia, headache, fever ≥38ºC, and epistaxis 1.3 to 2.1 times more (p>0.05). At the time of the survey, there were no statistically significant associations for seropositivity and clinical findings for the previous 3 months among those surveyed (p>0·05). A full list of clinical findings is illustrated in Figure 3.

Healthcare workers had a 1·7% (10/588) seroprevalence, while other sector workers had a 1·3% seroprevalence (46/3456). There were no statistically significant differences for seroprevalence between health sector and non-health sector population (p>0·05). In addition, known exposure to COVID-19 confirmed cases was not associated with seropositivity. Seropositive subjects experienced near two times higher hospitalisations within 3 months due to respiratory symptoms and signs compared to seronegative subjects (p>0.05). Concurrently, these subjects reported school or work misses and healthcare visits due to respiratory complaints 1·2 and 1·89 times more, respectively (p>0.05). Some risk factors and daily life disturbances due to SARS-CoV-2 are listed on Table 3. In addition, these variables did not result in significant associations with seropositivity in a multivariate logistic regression analysis with adjustments for age group and sex, (p>0·05).

**Table 3:** SARS-CoV-2 seropositivity and some associations of exposure and daily life disturbances, late 2020

		Positive		Negative		Prevalence Ratio	p value*	Adjusted Odd's Ratio	p value⁎⁎
		n	% (95%CI Lower-Upper bound)	n	% (95%CI Lower-Upper bound)	PR (95%CI Lower-Upper bound)		aOR (95%CI Lower-Upper bound)	
**Occupational type**						1·27 (0·65-2·52)	0·478	1·11 (0·41 -1·38)	0·808
Healthcare worker		10	1·70% (0·88-3·00)	578	98·30% (97·00-99·12)				
Non-health sector		46	1·33% (0·99-1·75)	3410	98·67% (98·25-99·01)				
**Contact with COVID-19 confirmed case**						1·02 (0·14-7·21)	0·988	1·26 (0·17-9·36)	0·820
Yes		1	1·45% (0·16-6·57)	68	98·55% (93·43-99·84)				
No		70	1·43% (1·12-1·79)	4834	98·57% (98·21-98·88)				
**Comorbidity (1 or more)**						0·96 (0·31-3·03)	0·949	0·82 (0·20-3·26)	0·774
Yes		3	1·39% (0·39-3·66)	213	98·61% (96·34-99·61)				
No		69	1·44% (1·13-1·81)	4715	98·56% (98·19-98·87)				
**Healthcare visit within last 3 months (due COVID-19 related symptoms and signs)**						1·89 (0·83-4·32)	0·125	1·81 (0·54-6·07)	0·335
Yes		6	2·62% (1·10-5·32)		97·38% (94·68-98·90)				
No		66	1·38% (1·08-1·75)		98·62% (98·25-98·92)				
**School or work miss within last 3 months (due COVID-19 related symptoms and signs)**						1·20 (0·17-8·49)	0·855	1·24 (0·12-12·81)	0·858
Yes		1	1·72% (0·19-7·78)		98·28% (92·22-99·81)				
No		71	1·44% (1·13-1·80)		98·56% (98·20-98·87)				
**Hospitalisation within last 3 months (due COVID-19 related symptoms and signs)**						2·17 (0·54-8·66)	0·265	0·76 (0·07-7·77)	0·816
Yes		2	3·08% (0·64-9·51)	63	96·92% (90·49-99·36)				
No		70	1·42% (1·12-1·78)	4864	98·58% (98·22-98·88)				

⁎ Chi-square test,

⁎⁎ Multivariate logistic regression analysis adjusted for sex and age group.

## Discussion

In this first nationwide seroepidemiological survey in Mongolia, 1·4% (95%CI; 1·2-1·7) of the study population from Mongolia could have been exposed to SARS-CoV-2 up to December 2020, despite reports in the RT-PCR testing-based sentinel surveillance system reporting no community transmission until November 10, 2020.^10^^,^^14^ We observed serologically positive subjects of SARS-CoV-2 in all subgroups of age, sex, and geographical location, even in the remotest and most dispersedly populated country neighbouring China, where the novel coronavirus is believed to have originated.^1^ Findings of the survey suggest a small scale yet evenly distributed outbreak could have occurred in Mongolia before the notification of the first local outbreak.

The evenly distributed, low seroprevalence level of SARS-CoV-2 antibodies in Mongolia could be due to several reasons. Throughout most of 2020, RT-PCR based surveillance could have missed COVID-19 cases leading to sporadic outbreaks that remained relatively well-contained due to preventive measures including mandatory mask wearing and school closures.^1^ However, this hypothesis remains unlikely since clinical samples showed no indications of local outbreaks. We expected that Ulaanbaatar city would have higher seroprevalence due to its population density and potential leaking from quarantine camps for all incomers by charter flights, yet the rural and urban population showed no difference in seropositivity. Despite this, border provinces including Bayan-Ulgii and Selenge had seropositive subjects where land transportation continued to import goods. Truck drivers could have been the likely source of small-scale SARS-CoV-2 outbreaks coupled with inadequate surveillance testing sampling size, particularly in rural areas.^1^ Another possibility could be an early introduction of SARS-CoV-2 in Mongolia circulating during the influenza season of late 2019, however least likely given that the Mongolian population remained isolated since March 10, 2020, and only people without travel histories were included in our analysis. Nevertheless, serum samples collected from September to December 2019 had a seropositive rate of 1·9% using IgG, compared to a rate of 12·4% using IgM in Italy.^1^ Lastly, cross-reactivity to other human coronaviruses could have occurred in our survey. In Hong Kong, similarly low level of 2% seropositivity among SARS-CoV-2 naïve individuals are reported.^1^

The low level of COVID-19 in our survey is also reflected in changes to other disease patterns that share similar transmission routes including acute respiratory infections. Respiratory illnesses were the leading cause of both outpatient and inpatient morbidity in Mongolia before the COVID-19 pandemic.^1^ The country of 3·3 million bears a huge burden of respiratory infections peaking seasonally in the colder months in Mongolia, coupled with ambient air pollution in Ulaanbaatar and the provincial centres. However, in 2020, reports of respiratory diseases (that could be undetected COVID-19) and related hospitalisations dropped dramatically by up to 2 times according to national health statistics.^1^ This decrease was due to various public health measures including the closure of educational facilities, early adoption of mask-wearing and large scale influenza vaccinations and lockdowns in later 2020. Avoiding unnecessary visits to clinical facilities was not extensively enforced until November, 2020 in Mongolia.^1^ In addition, local transmission of SARS-CoV-2 was at a low level as evidently Mongolia did not report excess deaths throughout 2020.^7^^,^^8^

This survey potentially included relatively older as well as new SARS-CoV-2 infections. Five samples turned negative on SARS-CoV-2 IgG quantitative assay that were initially positive to total antibodies to SARS-CoV-2. These samples may have been acute onset of infection with positive IgM or IgA. However, in these samples there were no statistically significant clinical findings of SARS-CoV-2. Seven positive cases that initially resulted negative to total antibodies may have had COVID-19 infection much earlier, as antibodies titre decrease naturally in some individuals.^1^

We established the baseline seroprevalence prior to vaccination with this survey. Additional serosurveys in Mongolia are needed to monitor progress towards herd immunity acquired through natural infection and vaccination to inform the national COVID-19 vaccination strategies. Knowledge on herd immunity will allow easing of movement restrictions and provide insight into the effectiveness of Mongolia's vaccination programme. Over 90% of the target population (adults) received at least a single dose of COVID-19 vaccine as of late June 2021, achieving one of the highest vaccination rates in the world and within the region.^1^ Despite this, the country has experienced a large number of cases proportional to its population in 2021.^1^ It is believed the first local transmission was the result of a poorly managed mandatory quarantine in November 2020. Since the local transmission, Mongolia has gone into nationwide and urban lockdown 4 times (as of August 2021), yet has been unable to fully control the spread.^1^ Two large waves peaked in late April and late June 2021, even though mask wearing and other preventive measures have been widely practiced.^1^ The high case counts despite vaccination and prevention measures suggest Mongolia remains vulnerable to wider spread of SARS-CoV-2 in this second year of the pandemic, especially having had rising reproduction numbers in the first half of 2021.^1^

Despite the excellent control of SARS-CoV-2 transmission in 2020, complications of such stringent policies are not to be dismissed. Firstly, socio-economic impacts were strenuous, especially to small business owners and the vulnerable, although the government supported in various ways.^1^ Secondly, in-class educational services for all levels have been disrupted substantially in 2020 and 2021.^5^^,^^17^ Thirdly, the health impacts of the pandemic other than SARS-CoV-2 infection are yet to be determined. The Mongolian population already had a high proportion of non-communicable disease risk factors, which may be exacerbated by people spending more time indoors.^1^ In 2015 and 2016, Mongolia experienced a large outbreak of measles after a short-lived measles eradication status, potentially due to the absence of an individually-tracked electronic record system for routine vaccination leading to an accumulation of unvaccinated individuals.^1^ Disruptions to routine immunisations could have occurred during lockdowns. Other negative impacts surrounding mental wellbeing and domestic violence were also reported in Mongolia, especially for women and girls.^1^

Our study has several limitations. Firstly, we did not explore the neutralising antibodies test for SARS-CoV-2 for definite positive case analyses and could not rule out for cross-reactivity. However, we did use confirmatory testing to minimise false positive results that would otherwise be common in a low prevalence setting such as this study. Secondly, IgM tests was not performed for positive samples with total antibody test and negative to SARS-CoV-2 IgG: anti-SP and anti-RBD test. The timeline of the survey includes a local outbreak free period in the rural provinces and the early onset of local transmission in Ulaanbaatar. As a result, acute onset infections could not be ruled out for samples from Ulaanbaatar that reported SARS-CoV-2 infections in November 2020. These limitations could potentially lead to overestimation of the seroprevalence. Among subjects, more females were selected leading to skewed distribution for sex. In addition, survey questions related to signs of acute respiratory infection in the previous three months are subject to potential recall bias.

Most countries and territories in the Western Pacific region maintained stringent policies to contain SARS-CoV-2, and overall, the region had lower cases and seroprevalence than the global average. [^2^,^23^,^24^] Mongolia had one of the lowest prevalence of SARS-CoV-2 in the first year of the pandemic, similar to other countries in the region such as Viet Nam and Taiwan Special Administrative Region.^3^^,^^23^ Policy actions including border closure, mandatory quarantine for incoming travellers and school closures potentially prevented widespread local transmission up to the end of 2020. The low level of seroprevalence in our 2020 survey may reflect the public health measures and stringent policies taken early in rapid response to a pandemic in a LMIC setting, preventing excess deaths before vaccines were produced.^1^ Future investigations should identify immunity as a result of vaccination and protective responses in Mongolia in order to maximise 2022 vaccination campaigns.

## Contributors

BC served as principial investigator for the project. UM, OE, KN, TD, DB, TT, DG, OD conducted planning and coordination. L-VL, NP, NP, LS has been supervisors. BB, ZD, OA, BM, AG, OB, GE, BS, MD, did data collection and assisted laboratory work. AA, KE, UsM, BB, TB, BD, MyD, ZN, KT, KT, EB, AB, YM, OS, MG, OB, BayB, BatzB, GZ, assisted data collection and conducted laboratory work. BB, GG, RE did data analysis. RE, BC, UM, BB, GG, ZD came up with first manuscript. Writing and conceptualisation has been conducted by RE, UM and BC. L-VL, LS, BV, IB conducted manuscript editing and revisions. GG did English proofreading.

## Declaration of Competing Interest

None.

## Appendix Supplementary materials

Image, application 1

Image, application 2

Image, application 3

Image, application 4
