# Supplementary material for: SARS-CoV-2 seroprevalence in Mongolia: Results from a national population survey
Source: Lancet Reg Health West Pac. 2021 Nov 23;17:100317. doi: 10.1016/j.lanwpc.2021.100317 (PMC8609908; doi:10.1016/j.lanwpc.2021.100317)
Supplement: Supplementary file 1 [file mmc1.pdf]

Population-based age-stratified sero-epidemiological investigation protocol for COVID-19 infection

Novel Coronavirus (COVID-19) Population-Based Age-Stratified Sero-Epidemiological Study in Mongolia (A Prospective, Nationwide Study)

1. **Project Title:** Novel Coronavirus (COVID-19) population-based age-stratified sero-epidemiological study in Mongolia (A Prospective, Nationwide Study)
2. **Sub Area:** Population-based age-stratified sero-epidemiological investigation protocol for COVID-19 infection
3. **Duration in months:** 12 months
4. **Project Category:** Applied Research

   Any other: NA

5. **Participating organizations:**

   Ministry of Health Mongolia

   Mongolian National University of Medical Sciences

   The World Health Organization

6. **Principal Investigator:**

   Assoc.Prof Battogtokh Chimeddorj, MD, PhD

   Head of Department of Microbiology and Infection Prevention Control, School of Biomedicine, Mongolian National University of Mongolia

7. **Advisors:**

   Academician Nyamadawa P, MD, ScD, General Virologist, Professional Committee for Virology, Ministry of Health, Mongolia and Advisor for the

National Influenza Center

Professor Davaalkham D, MD, PhD, Dean School of Public Health, MNUMS

Dr Linh-Vi LE, PhD, MSc, MA, Epidemiologist, WPRO, WHO

8. Co-Investigators:

Professor Khurelbaatar N, MD, PhD, President, Mongolian National University of Medical Sciences (MNUMS)

Damdindorj B, MD, PhD, Vice President for Research and International relation, Mongolian National University of Medical Sciences

Darambazar G, MD, PhD, Dean, School of Biomedicine, MNUMS

Dr Oyunsuren E, MD, PhD, Senior Expert, Ministry of Health, Mongolia

Assoc Professor Tuvshinjargal Ts, MD, PhD, Dean, Mongolian National University of Medical Sciences
Dr Undram, MD, PhD, Senior Lecturer, Department of Family Medicine, School of Medicine, Mongolian National University of Medical Sciences

Khongorzul T, MD, PhD, Head, Department of Immunology, School of Biomedicine, MNUMS

Ariunzaya B, MD, PhD, Department of Immunology, School of Biomedicine, MNUMS

Zolmunkh N, MD, PhD, Department of Immunology, School of Biomedicine, MNUMS

Enkh-Amar B, MD, Department of Immunology, School of Biomedicine, MNUMS

Otgonjargal B, PhD, Department of Microbiology and Infection Prevention Control, School of Biomedicine, MNUMS

Battur M, MSc, Department of Microbiology and Infection Prevention Control, School of Biomedicine, MNUMS

Oyunbaatar A, MSc, Department of Microbiology and Infection Prevention Control, School of Biomedicine, MNUMS

Zolzaya D, MD, MSc, Department of Microbiology and Infection Prevention Control, School of Biomedicine, MNUMS

Anuujin G, MD, MSc, Department of Microbiology and Infection Prevention Control, School of Biomedicine, MNUMS

Batzaya B, MSc, Department of Laboratory, Microbiology section, Mongolia-Japan University Hospital, MNUMS

Gereltsetseg Z, Department of Department of Laboratory, Immunology section, Mongolia-Japan University Hospital, MNUMS

Batzorig B, PhD, Department of Epidemiology and Biostatistics, School of Public Health

Oyungerel D, MSc in Public Health, Department of Epidemiology and Biostatistics, School of Public Health

Mandukhai G, MSc in Public Health, Department of Epidemiology and Biostatistics, School of Public Health

Erkebulan M, MSc, Department of Epidemiology and Biostatistics, School of Public Health

Khangai E, MSc, Department of Family Medicine, School of Medicine
Batkhuu B, MSc, Department of Family Medicine, School of Medicine

Tuyajargal.B, MSc, Department of Family Medicine, School of

Medicine

Usukhbayar. M, MSc, Department of Family Medicine, School of Medicine
Assoc.Prof Myagmartseren D, MD, PhD, Department of Family Medicine, School of Medicine

Dr Roger Evans, Laboratory virologist, WPRO, WHO

Dr Ariuntuya, MD, TO, WHE, WHO Mongolia CO

Summary

Novel Coronavirus (COVID-19) population-based age-stratified sero-epidemiological study in Mongolia (A Prospective, Nationwide Study)	
**Study population**	Age-stratified random sample from general population
**Potential output and analysis**	Estimate or inform estimates of:<br>• Seroprevalence of antibodies to COVID-19<br>• Cumulative incidence of infection<br>• Infection attack rates<br>• Fraction of asymptomatic infection<br>• Case fatality ratio
**Study design**	Prospective population-based convenience sample from the general population, stratified by age<br>• Longitudinal cohort investigation with serial sampling of the same individuals
**Study duration**	Serial sampling as a prospective cohort study<br>Total duration: **1 year**
**Minimum information and specimens to be obtained from participants**	Data collection: Epidemiological data including basic demographics and clinical symptoms<br>Specimens: Serum samples to inform sero-epidemiological inferences

1. Background

Mongolia's surveillance system for COVID-19 currently consists of reporting of case detection. As of 31 August 2020, there were 301 cases of COVID-19 detected and no deaths in Mongolia. Despite the low number of cases, there is potential for increases in cases when public health and social measures are relaxed and given that Mongolia shares a border with China, where the SARS-CoV-2 virus was first detected in Wuhan city in December 2019. In support of the Ministry of Health's decision to increase understanding on the extent and characteristics of SARS-CoV-2 infection in the population of Mongolia, the National University of Mongolia and partners propose to adapt the UNITY early sero-epidemiological investigation protocols[1].

As is the case with SARS-CoV-2, the detection and spread of an emerging respiratory pathogen are accompanied by uncertainty over the key epidemiological and serologic characteristics of the novel pathogen and particularly its transmissibility (i.e. ability to spread in a population) and its virulence (i.e. case-severity). To date initial surveillance has focused primarily on patients with symptoms or severe disease, and, as such, the full spectrum of the disease, including the extent and fraction of mild or asymptomatic infections that do not require medical attention are not clear. Estimates of the case fatality ratio, and other epidemiological parameters, will likely be lower than current estimates once the full spectrum of disease is able to be included in the denominator. In addition, the role of pre- symptomatic, asymptomatic or subclinical infections in human-to-human transmission of SARS-CoV-2 virus is not well understood.

With a novel coronavirus, initial seroprevalence in the population is assumed to be negligible due to the virus being novel in origin. Therefore, surveillance of antibody seropositivity in a population can allow inferences to be made about the extent of infection and about the cumulative incidence of infection in the population.

The proposed study will investigate the extent of infection, as determined by sero-positivity in the general population nationwide in Mongolia. We have tailored some aspects of the UNITY protocol to align with public health, laboratory and clinical systems

[1] https://www.who.int/emergencies/diseases/novel-coronavirus-2019/technical-guidance/early-investig

in Mongolia and according to capacity, availability of resources and cultural appropriateness. By applying WHO's standardized protocol and using testing kits provided by WHO, our data would be comparable to other countries. We will contribute aggregated data on seroprevalence and epidemiological exposures to WHO UNITY studies for regional and global analyses across many different settings for timely estimates of COVID-19 virus infection, severity and attack rates, as well as to inform public health responses and policy decisions. This is particularly important in the context of a novel respiratory pathogen, such as COVID-19.

2. Study objectives

The objectives for this sero-epidemiological investigation are as follows:

1. To measure the seroprevalence of antibodies to COVID-19 in the general population at urban, rural and tribal areas by sex and age groups across Mongolia.
2. To estimate the fraction of asymptomatic, pre-symptomatic or subclinical infections in the population and by sex and age group.
3. To determine risk factors for infection by comparing the exposures of infected and non- infected individuals.
4. To contribute to determine the case fatality ratio.
5. To contribute to an improved understanding of antibody kinetics following COVID-19 infection.

Note: Little is currently known about COVID-19 virus antibody kinetics. Asymptomatic infected persons may clear the virus more quickly than do symptomatic patients. Antibody titers in the asymptomatic persons are likely to be lower, if they seroconvert at all, than in infected patients exhibiting symptoms. These are considerations for the interpretation of any COVID-19 virus sero-epidemiological investigation. We will be taking this aspect into consideration.

3. Methods

Study design

The sero-epidemiological investigation for COVID-19 virus infection is a **population-based, age- stratified prospective study**. It is intended to provide key epidemiological and serologic characteristics of SARS-CoV-2 virus in Mongolia.

Through UNITY, the WHO aims to gather data from countries around world; we will be contributing from Mongolia data from longitudinal follow-up of a cohort of randomly selected participants to increase understanding of various unknown facts about antibody kinetics in symptomatic and asymptomatic, incidence, attack rates, case fatality, and determinants of infection. The detection of seroprevalence over time in Mongolia will help inform the extent of infection in the community and the level of individuals susceptible to SARS-CoV-2 over time.

We will conduct a longitudinal cohort investigation with serial sampling of the same individuals over four periods of time (every quarter).

We will take the first sample for sero-positivity after collecting all baseline information on Day 1.

Follow-up samples from the same individuals will be repeated at months 3, 6 and 9.

Repeated samples at 3, 6 and 9 months would help us to calculate cumulative incidence of sero-conversion in population at various sites in different age groups.

The follow-up of positives will enable us to understand antibody kinetics following COVID-19 infection.

Sampling strategy and selection of clusters

In order to collect baseline data in the shortest possible time, we will be focusing on getting primary data within 2 months.

Sampling method

Three stage sampling (major cities, provincial centers and soum) was performed to cover all socio and geographical regions of Mongolia. (Figure 1)

In the next stage, multistage randomized sampling was performed to select study participants. Overall, 5000 participants were divided into 50 clusters, and were calculated as 100 participants to be selected from each cluster. Based on the population density, a total of 27 clusters selected from aimags and 23 clusters from Ulaanbaatar city. At aimag level, one cluster selected from provincial center and 2 clusters from soums in each aimag.

For each cluster, potential participants will be selected from rosters at health centers, which consist of all residents in the cluster area. To include all age groups in the community, we will select study participants from local residents by the age stratified random sampling method.

Fig.1 Multi-stage random sampling scheme

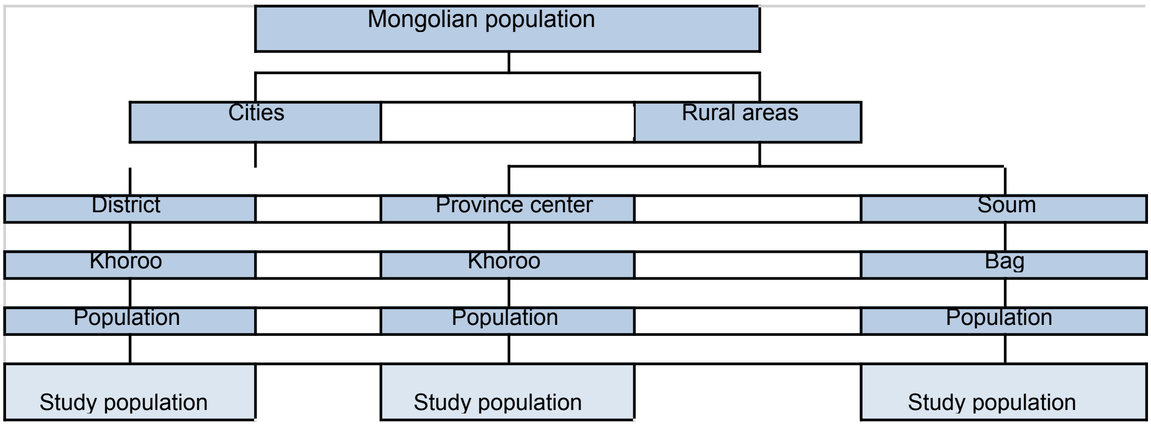

Sample size

The sample size is calculated for an expected 1% prevalence of SARS-CoV-2 antibodies with a 0.4% margin of error and design effect of 2 (see below sample size calculation). Although the sample size is calculated using a prevalence of 1%, given the current low case reports, we expect a prevalence of <1%. The target sample size would enable detection of seroconversion to obtain incidence. Table 1 shows study clusters from each region and capital city.

Sample size calculation

$$n = \frac{DEFF * N * Z^2 * p * (1-p)}{(N-1) * e^2 + Z^2 * p * (1-p)} = \frac{2 * 3238479 * 1.96^2 * 1.0 * 99.0}{3238478 * 0.4^2 + 1.96^2 * 1 * 99} = 4750$$

- DEFF=2.0 /Design effect /
- N = 3 238 479 /Number of General Population of Mongolia, reported by National Statistical Organization, 2018/
- Z = 1.96 /critical value of standard normal distribution/
- P = 1% /expected sero-prevalence of antibodies to SARS-CoV-2,
    - *Havers FP, Reed C, Lim T, et al. Seroprevalence of Antibodies to SARS-CoV-2 in 10 Sites in the United States, March 23-May 12, 2020.* JAMA Intern Med. *Published online July 21, 2020. doi:10.1001/jamainternmed.2020.4130/*
- e = 0.4 /margin of errors/

This would be done in an attempt to include participants **over a range of ages** in order to determine and compare age-specific sero-prevalence. This will ensure that the following 10 age groups are reported: 1-4, 5-9, 10-14, 15-19, 20-29, 30-39, 40-49, 50-59, 60-69, 70+. Sampling within each stratum will be done by systematic sampling.

Table 1. Age group fraction of the study population

Age, years	Number of Total Population	Fraction	Sample size by age groups
0-4 yrs	391,125.	12	600
5-9 yrs	358,379.	11	550
10-14 yrs	252,548.	8	400
15-19 yrs	228,402.	7	350
20-29 yrs	539,521.	17	850
30-39 yrs	539,323.	17	850
40-49 yrs	413,694.	13	650
0-59 yrs	299,756.	9	450
60-69 yrs	138,097.	4	200
70 +yrs	77,634.	2	100
Total	3,238,479.		5000

We propose to take a population of 600 or 900 depending on the population size in the region and 2300 in the capital city where 60% of the Mongolian population resides, totaling 5000 samples.

Table 2. Number of Clusters selected from each provinces and Ulaanbaatar city and sample size of the study

Region	Selected provinces/ and Districts	Number of clusters			Sample size
		In a province center	In a soum	Total	
Eastern region	Dornod aimag	1	2	3	300
	Khentii aimag	1	2	3	300
**Sub total**				**6**	**600**
Central region	Selenge aimag	1	2	3	300
	Umnugobi aimag	1	2	3	300
	Dornogobi aimag	1	2	3	300
**Sub total**				**9**	**900**
Khangai region	Erdenet city	1	2	3	300
	Bayankhongor aimag	1	2	3	300
**Sub total**				**6**	**600**
Western region	Bayan-Ulgii aimag	1	2	3	300
	Zavkhan aimag	1	2	3	300
**Sub t otal**				**6**	**600**
Capital city, districts	Songinokhairkhan district	5		5	500
	Khan-Uul district	3		3	300
	Bayangol district	4		4	400
	Bayanzurkh district	4		4	400
	Chingeltei district	4		4	400
	Sukhbaatar district	3		3	300
				**23**	**2300**
**TOTAL**				**50**	**5000**

Study population

Selection of provinces and cities:

Mongolia is geographically divided into 5 regions: West, Khangai, Central, East, and Ulaanbaatar based on the residential area of population.

All 21 provinces (aimags) of Mongolia are listed below by region:

- Western region provinces include: Bayan-Ulgi (1), Gobi Altai (2), Zavkhan (3), Khovd (4), Uvs (5)

- Khangai region provinces include: Arkhangai (1), Bayankhongor (2), Bulgan (3), Uvurkhangai (4), Khuvsgul (5)
- Central region provinces include: Umnugobi (1), Dornogobi (2), Dundgobi (3), Gobisumber (4), Tuv (5), Selenge (6), Darkhan-Uul (7), Orkhon (8)
- Eastern region provinces include: Khentii (1), Dornod (2), Sukhbaatar (3)

From above 21 provinces Two provinces from each region and Ulaanbaatar city were selected randomly by using the “randbetween” tool in MS Excel.

The study population will be citizens and randomly selected from the following provinces (8) and cities (2):
Dornod, Khentii, Umnugobi, Dornogovi, Selenge, Bayankhongor, Bayan-Ulgii and Zavkhan aimags and Erdenet, Ulaanbaatar cities (selected provinces marked with star in Figure 2). These 9 areas cover almost half of the 21 provinces of Mongolia and represent both high and low incidence areas along with urban and rural populations of Mongolia. Additionally, except Ulaanbaatar city, Mongolia has only two minor cities: Darkhan and Erdenet. From these two cities, Erdenet was also selected randomly.

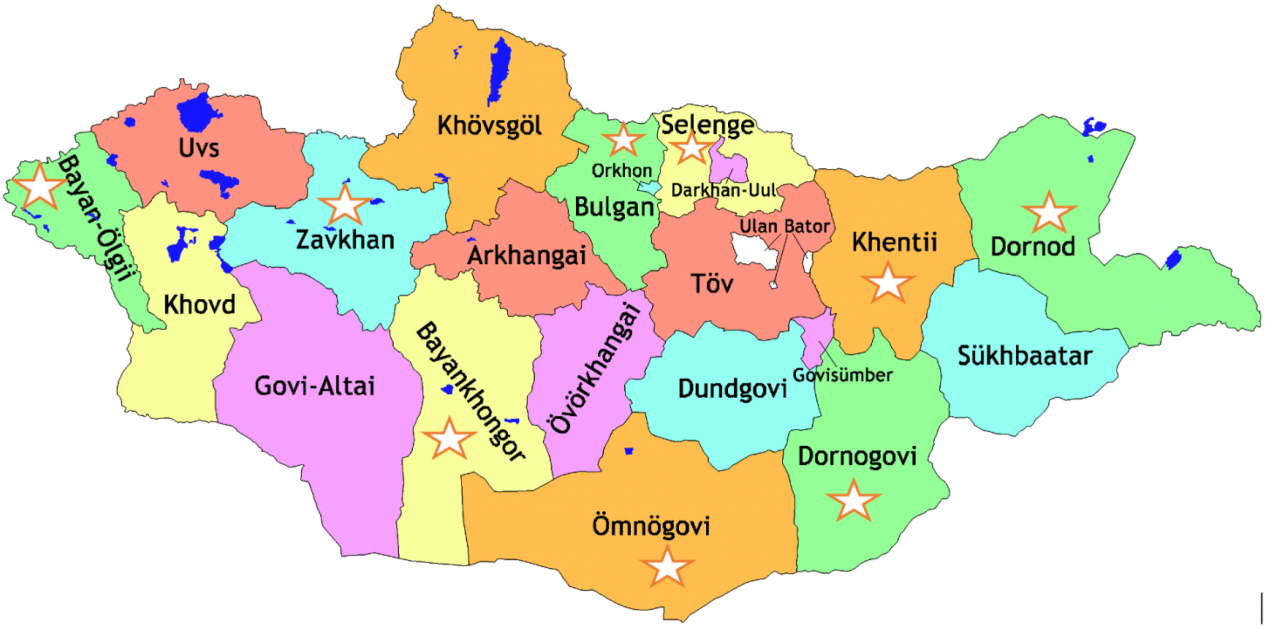

Fig 4. Selected provinces for the survey

Ulaanbaatar ‘s team (working group) will be the central coordinating site for all other

nine study sites which will provide overall technical support, guidance and will coordinate together with a regional team.

Table 3. Soum and khoroo level selection

Sites	Provinces	Selected soums and khoroos
Eastern site	Dornod aimag	Province center: Kherlen 1st khoroo,<br>Soums: Tsagaan Ovoo, Bayandun
	Khentii aimag	Province center: 2nd khoroo,<br>Soums: Batnorov, Bayankhutag
**Total=600**		
Central site	Selenge aimag	Province center: 1st and 3rd khoroo<br>Soums: Altanbulag, Khushaat
	Umnugobi aimag	Province center: 2nd khoroo<br>Soums: Khankhongor, Khurmen
	Dornogobi aimag	Province center: 1st khoroo<br>Soums: Urgun, Zamiin uud
**Total=900**		
Khangai sites	Erdenet city	Province center: 1st and 3rd khoroo<br>Soums: Jargalant
	Bayankhongor aimag	Province center: 1st khoroo,<br>Soums: Bogd, Jinst
**Total=600**		
Western sites	Bayan –Ulgii	Province center: 1st khoroo<br>Soums: Sagsai, Ulaankhus
	Zavkhan aimag	Province center: 2nd khoroo<br>Soums – Tsagaankhairkhan , Aldarkhaan
**Total=600**		
Capital city	Songinokhairkhan district	Khoroo-9, 19, 17, 1, 12
	Khan-Uul district	Khoroo-1, 5, 13
	Bayangol district	Khoroo-3, 13, 16, 20
	Bayanzurkh district	Khoroo-2, 13, 20, 24
	Chingeltei district	Khoroo-1, 6, 14, 19
	Sukhbaatar district	Khoroo-1,11,17
**Total=2300**		
**Grand total=5000**		

Epidemiological indicators

The table below provides an overview of the epidemiological parameters that can be measured as part of this investigation.

Table 4: Indicators to inform investigation objectives

Objective	Parameter	Definition	Data source to calculate the parameters concerned	Comments, limitations
**1. Measure the sero-prevalence of antibodies to COVID-19 in the general population by age group** in order to ascertain the cumulative population immunity	Sero-prevalence (population and age-specific)	The proportion of individuals per age strata who show sero-positivity for COVID-19 virus infection	- Seropositivity<br>- Age group	- Population seroprevalence will be calculated using direct standardization methods, so that the proportion is adjusted for any difference in the age stratification of the participants and the overall population<br>- Age-specific seroprevalence is same as ***age-specific attack rate*** and ***cumulative incidence*****.**<br>- If data is collected, seroprevalence by different groups (e.g. geography, profession, residence) will be an important sub-analysis depending on numbers we get.

**2. Estimate the fraction of asymptomatic or pre-symptomatic/ subclinical infections in the population and by age group.**	Asymptomatic Fraction (proportion of cases that are asymptomatic)	The proportion of individuals who reported no symptoms of COVID-19 infection of individuals seropositive for COVID-19	- Sero- positivity<br>- Reported symptom s	- The numerator is the number of individuals reporting no symptoms and the denominator is the total number of individual's sero-positive for COVID-19.
	Fraction severe disease	The number individuals with severe infection	- Sero-positivity<br>- Reported symptoms<br>- Age group	- Severe disease will be defined (like hospitalization).<br>- The number individuals with severe infection divided by the number with COVID-19 infection as determined by sero-positivity.
**3. Determine risk factors for infection** by comparing the exposures of infected and non-infected individuals	Population groups most at risk	The identification of groups who are most vulnerable to COVID-19 virus infection (e.g. age groups, gender, occupation)	- Sero-positivity<br>- Reported symptom s<br>- Exposure of interest (e.g. age group)	- May only be an early signal, a nested case-control study could be conducted to evaluate risk factors for infection if required.
**4. Contribute to determine the case fatality ratio**	Case fatality ratio	The proportion of individuals with fatal outcome for COVID-19 Infection	- Sero-positivity<br>- Mortality Age group	- We will use longitudinal cohort investigations to record sufficient events (i.e. deaths)<br>- May require extended follow-up to determine outcome of those with COVID-19 infection beyond 2 years if required at that time for long term sequelae
**5. To contribute to an improved understanding of antibody kinetics following COVID-19 infection.**	Serological response to infection	The change in serum level of specific antibodies to COVID-19 virus (*Increase in titer*)	- Antibody titer	- Changes in titers would be calculated using geometric mean titers (GMTs) and PRNT

We will follow all positives every three months for nine months to see the antibody titers for antibody kinetics. **(Fig. 3)**

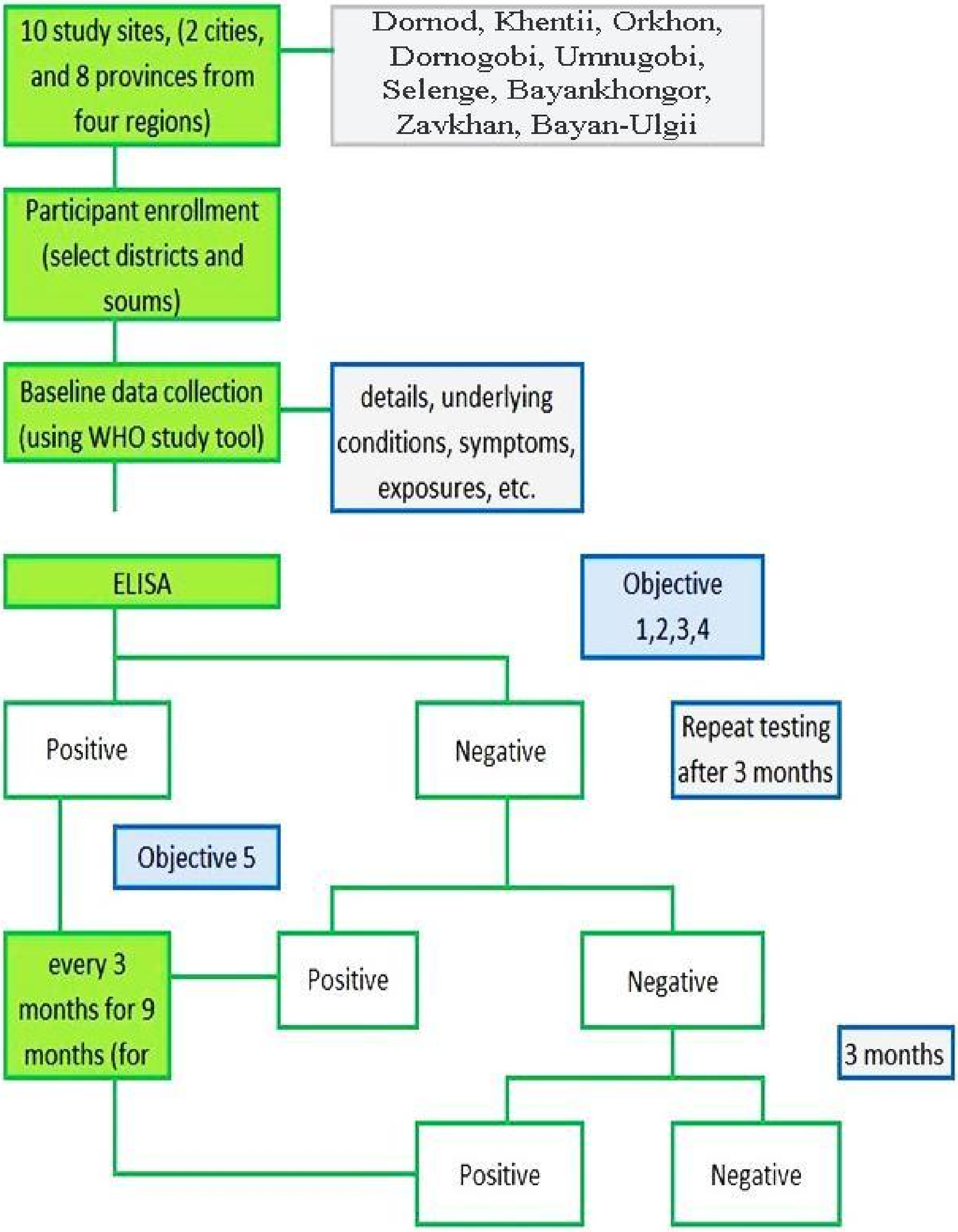

Fig 3: Case investigation algorithm and summary of data-collection tool

Samples will be drawn and would be transported, stored and tested as per standard protocol of the test kit used. Positive samples will be collected every three months for at least 9 months to see persistence of antibody titer. We will be doing the ELISA test

at Ulaanbaatar University hospital laboratory.

[1] Riley S, Kwok KO, Wu KM et al. Epidemiological characteristics of 2009 (H1N1) pandemic influenza based on paired sera from a longitudinal community cohort study. *PLoS Med*. 2011 Jun;8(6):e1000442

Eligibility criteria

Inclusion criteria:

All individuals identified for recruitment into the investigation, irrespective of age, irrespective of acute or prior COVID-19 infection.

Exclusion criteria:

Refusal to give informed consent, or contraindication to venipuncture

The confirmed cases only imported by international charter flight

Data and specimen collection and transport

Information about the subject's age, gender, area of residence, occupation, workplace; number of persons in the household, travel, exposure details, and (symptoms of respiratory illness if any, duration of disease, medical consultation, treatment, hospitalization and outcome) as per WHO protocol will be collected using a paper based/ tablet-based study tool. We would be contributing this data to global data from UNITY studies.

Data and specimen collection will be repeated in selected individuals who were participants with negative results at baseline will be followed up after 3 months, and if further negative, followed up again at another 3 months (total number of samples taken will be 3, at months 0, 3 and 6). If the participant seroconverts at any of the time periods, they will be followed up for one year (total number of samples depends on when seroconversion takes place, at minimum if the result is positive at baseline, then follow- up will be months 3, 6 and 9 for a total of 4 samples taken), and then in negatives at 6 months. Longitudinal follow-up of positive cases will be conducted to understand immunity kinetics.

Data and specimen collection at each site will be conducted in primary health care settings (family and soum health care center). To cover the unreached population in remote areas data and specimen collection will be conducted in households.

Each participant recruited and sampled for the investigation would be asked to complete a questionnaire which covers demographic, clinical and exposure information. An example of an investigation questionnaire which will be used can be found in the Appendix: *Form 1 "Reporting form for each participant"*.

The data collection also includes the reported COVID-19 laboratory testing by investigation subjects.

A serum sample will be collected from each participant upon recruitment into the investigation. Totally, 5 ml blood from adults and 3 ml blood from children will be collected each time and will make 3 aliquots for lab testing. All those involved in the collection and transportation of specimens will be trained in safe handling practices and spill decontamination procedures. For details regarding the transport of samples collected and infection control advice, we will follow case management algorithm and laboratory guidance in the country or WHO laboratory guidance, available on the WHO website.

For each biological sample collected, the time of collection, the conditions for transportation and the time of arrival at the study laboratory will be recorded. Specimens will be transported in a portable refrigerator for sample transportation and reach the laboratory as soon as possible after collection. In case of need to store specimens in provincial and soum level, medical freezers of soum and family health centers will be used. All soums selected to the study have capacity of centralized electricity. In case of electricity off, all soums have portable generators.

Serum will be separated from whole blood and transported at 4°C or frozen to -20°C or lower (at -80°C) and will be shipped on dry ice if required.

Laboratory procedures

Laboratory procedures involving sample manipulation will be carried out in a bio-safety cabinet (BSC) in the University hospital laboratory.

Serum samples will be screened for the presence of COVID-19 virus specific antibodies using serological testing. Tests for total antibodies for SARS-CoV-2 will be tested by Wantai Total Ab ELISA kit and Florian Krammer's ELISA from Kantaro (New York) ELISA kits. The Kantaro kit's a 2 step ELISA, 1st on RBD antigen and then the positives are tested against the spike antigen which is WHO procured.

ELISA testing would be carried out in a facility with **at bio-safety level 2 (BSL-2)** capacity. Serum samples will be stored at -80°C. Neutralizing antibody testing will not be applied as there are no facilities available in Mongolia. Stored specimens may be tested in the future should tests become available for detecting neutralizing antibodies by ELISA. We would **aliquot** samples prior to freezing, to minimize freeze thaw cycles.
The storage of serum specimens in domestic frost-free freezers should be avoided, owing to their wide temperature fluctuations.

Ethical considerations

Ethical clearance will be obtained from the Bio-Ethics and Research Committee, MOH before the start of study.

Informed consent

The purpose of the investigation will be explained to all individuals identified for recruitment into the investigation. Written informed consent will be obtained from all individuals willing to participate in the investigation before any procedure is performed as part of the investigation by a trained member of the investigation team. Consent for children under the legal age of consent will be obtained from a parent or legal guardian. Each participant must be informed that participation in the investigation is voluntary and that s/he is free to withdraw, without justification, from the investigation at any time without consequences and without affecting professional responsibilities.
Informed consent will seek approval to collect blood and epidemiological data for the intended purpose of this investigation, that samples may be shipped outside of the country for additional testing and that samples may be used for future research purposes. *Risks and benefits for subjects*

This investigation poses minimal risk to participants, involving the collection of small amounts (3 ml in adults, 2 ml in children) of blood. The primary benefit of the study is

indirect in that data collected will help improve and guide efforts to understand extent of COVID-19 virus infection and may prevent further transmission of the virus.

Confidentiality

Participant confidentiality will be maintained throughout the investigation. All subjects who participate in the investigation will be assigned a study identification number by the investigation team for the labeling of questionnaires and specimens. The link of this identification number to individuals will be maintained by the investigation team and the Ministry of Health (or equivalent) and will not be disclosed elsewhere.

If the data is shared by the implementing organization to WHO or any agency or institution providing support for data analysis, data shared will include only the study identification number and not any personal identifiable information.

Prevention of COVID-19 infection among study personnel

All personnel involved in the investigation would be trained in infection prevention and control procedures (standard contact and droplet precautions, as determined by national guidelines). These procedures should include proper hand hygiene and the correct use of surgical masks, if necessary, not only to minimize their own risk of infection when in close contact with individuals with COVID-19 infection, but also to minimize the risk of spread among other participants in the investigation.

Timeline

Activities	0-1 mo	1mo	3-4 mo	6-7 mo	9-10 mo	11-12 mo
• Protocol review meeting and ethical clearance<br>• Team recruitment, sensitization and trainings						
• Field visits, participant enrolment<br>• Data collection, blood sampling<br>• Database creation, data entry						
• 2nd round of data collection (for Incidence)<br>• Field visits, participant enrolment<br>• Data collection, blood sampling, data entry<br>• Follow-up of positive cases for objective 4 and 5						
**Midterm analysis**						
• 3rd round of data (for Incidence)<br>• Field d visits, participant enrolment<br>• Data collection, blood sampling, data entry<br>• Follow-up of positive cases for objective 4 and 5						
• 4th round of data (for Incidence)<br>• Field d visits, participant enrolment<br>• Data collection, blood sampling, data entry<br>• Follow-up of positive cases for objective 4 and 5						
**INTERIM ANALYSIS**						
• All reports and final data handover						

4 References

WHO Situation reports
https://www.who.int/emergencies/diseases/novel-coronavirus-2019/situation-reports/

The Unity studies: early investigations protocols
https://www.who.int/emergencies/diseases/novel-coronavirus-2019/technical-guidance/early- investigations

Surveillance, rapid response team and case definitions
https://www.who.int/emergencies/diseases/novel-coronavirus-2019/technical-guidance/surveillance-and-case-definitions

Laboratory
https://www.who.int/emergencies/diseases/novel-coronavirus-2019/technical-guidance/laboratory-guidance

Clinical care
https://www.who.int/emergencies/diseases/novel-coronavirus-2019/technical-guidance/patient- management

Infection prevention and control / WASH
https://www.who.int/emergencies/diseases/novel-coronavirus-2019/technical-guidance/infection- prevention-and-control

Risk communications and community engagement
https://www.who.int/emergencies/diseases/novel-coronavirus-2019/technical-guidance/risk- communication-and-community-engagement

Examples of sero-epidemiological studies
https://www.medrxiv.org/content/10.1101/2020.04.19.20071563v1
https://www.medrxiv.org/content/10.1101/2020.04.13.20060467v1
https://www.medrxiv.org/content/10.1101/2020.04.14.20062463v1

WHO Scientific brief "Immunity passports" in the context of COVID-19
https://www.who.int/publications-detail/immunity-passports-in-the-context-of-covid-19

Appendix: Questionnaires

Population-based age-stratified sero-epidemiological investigation protocol for COVID-19 infection

Form 1: Reporting form for each participant

Unique ID	

**Current status**
□ Alive □ Dead □ Unknown/lost to follow-up

**1. Data Collector Information**	
Name of data collector	
Data collector Institution	
Data collector telephone number	
Mobile number	
Email	
Form completion date(dd/mm/yyyy)	___/_/_
Date of interview with informant (dd/mm/yyyy)	___/_/_

**2. Identifier information**	
First name	
Family name	
Sex	□ Male □ Female □ Not known
Date of birth (dd/mm/yyyy)	___/___/____<br>□ Unknown
Age (years, months)	
Telephone (mobile) number	
Email	
Country of residence	
Nationality	
Ethnicity (optional)	
Occupation	□ HCW (including nursery and quarantine facility)<br>if yes, please write in detail ____________
	And please complete Form 3<br><br>□Non HCW<br>if yes, please write in detail ____________

Have you had contact with a anyone with suspected or confirmed COVID-19 virus infection?	□ Yes □ No □ Unknown<br><br>If Yes, dates of last contact (DD/MM/YYYY):<br>___ /_/_

3. Symptom history	
In the past (…) months, have you had any of the following:	
Fever (≥38 °C) or history of fever	□ Yes □ No □ Unknown
Sore throat	□ Yes □ No □ Unknown
Runny nose (rhinorrea)	□ Yes □ No □ Unknown
Cough	□ Yes □ No □ Unknown
Shortness of breath (dyspnea)	□ Yes □ No □ Unknown
Other respiratory symptoms	□ Yes □ No □ Unknown<br>If Yes, specify:
Chills	□ Yes □ No □ Unknown
Vomiting	□ Yes □ No □ Unknown
Nausea	□ Yes □ No □ Unknown
Diarrhea	□ Yes □ No □ Unknown
Headache	□ Yes □ No □ Unknown
Rash	□ Yes □ No □ Unknown
Conjunctivitis	□ Yes □ No □ Unknown
Muscle aches	□ Yes □ No □ Unknown
Joint ache(myalgia)	□ Yes □ No □ Unknown
Loss of appetite	□ Yes □ No □ Unknown
Loss of smell (anosmia)	□ Yes □ No □ Unknown
Loss of taste (ageusia)	□ Yes □ No □ Unknown
Nose bleed	□ Yes □ No □ Unknown
Fatigue	□ Yes □ No □ Unknown
Seizures	□ Yes □ No □ Unknown
Altered consciousness	□ Yes □ No □ Unknown
Other neurological signs	□ Yes □ No □ Unknown<br>If Yes, specify:
Other symptoms	□ Yes □ No □ Unknown<br>If Yes, specify:

4. Patient symptoms: complications	
Did any of these symptoms require you to seek medical attention?	□ Yes □ No □ Unknown
Did any of these symptoms require you to miss work or school?	□ Yes □ No □ Unknown
Hospitalization: Did any of these symptoms require you to be hospitalized?	□ Yes □ No □ Unknown

Form 2: Assessment of potential risk factors for coronavirus disease 2019 (COVID-19) among health workers in a health care setting

1. Adherence to infection prevention and control (IPC) measures information	
Smoker	□ Yes □ No
Occupation in health care facility	□ Medical doctor<br>□ Registered nurse (or equivalent)<br>□ Assistant nurse, nurse technician (or equivalent)<br>□ Radiology/x-ray technician<br>□ Phlebotomist<br>□ Physical therapist<br>□ Nutritionist/dietitian<br>**Other health personnel**:<br>□ Laboratory personnel<br>□ Admission/reception clerks<br>□ Patient transporters<br>□ Catering staff<br>□ Cleaners

2. Adherence to infection prevention and control (IPC) measures information	
What was the date of your most recent IPC training within the health care facility (dd/mm/yyyy)	___/___/___
How much cumulative IPC training (standard precautions, additional precautions) have you had at this health care facility	□ Less than 2 hours<br>□ More than 2 hours
Do you follow recommended hand hygiene practices?	□ Always, as recommended<br>□ Most of the time<br>□ Occasionally<br>□ Rarely
Do you use alcohol-based hand rub or soap and water before touching a patient?	□ Always, as recommended<br>□ Most of the time<br>□ Occasionally<br>□ Rarely
Do you use alcohol-based hand rub or soap and water before cleaning/aseptic procedures?	□ Always, as recommended<br>□ Most of the time<br>□ Occasionally<br>□ Rarely
Do you use alcohol-based hand rub or soap and water after (risk of) body fluid exposure?	□ Always, as recommended<br>□ Most of the time<br>□ Occasionally<br>□ Rarely
Do you use alcohol-based hand rub or soap and water after touching a patient?	□ Always, as recommended<br>□ Most of the time<br>□ Occasionally<br>□ Rarely
Do you use alcohol-based hand rub or soap and water after touching a patient's surroundings?	□ Always, as recommended<br>□ Most of the time<br>□ Occasionally<br>□ Rarely

Do you follow IPC standard precautions when in contact with any patient?	□ Always, as recommended<br>□ Most of the time<br>□ Occasionally<br>□ Rarely<br>□ I don’t know what IPC standard precautions are
Do you wear PPE when indicated?<br><br>(PPE includes: medical mask, face shield, gloves, goggles/glasses, gown, coverall, head cover, respirator (for example, N95 or equivalent) and shoe covers)	□ Always, according to the risk assessment<br>□ Most of the time, according to the risk assessment<br>□ Occasionally<br>□ Rarely
Is PPE available in sufficient quantity in the health care facility?	□ Yes □ No □ Unknown
