# Supplementary material for: SARS-CoV-2 seroprevalence in Mongolia: Results from a national population survey
Source: Lancet Reg Health West Pac. 2021 Nov 23;17:100317. doi: 10.1016/j.lanwpc.2021.100317 (PMC8609908; doi:10.1016/j.lanwpc.2021.100317)
Supplement: Supplementary file 2 [file mmc2.pptx]

## Slide 1
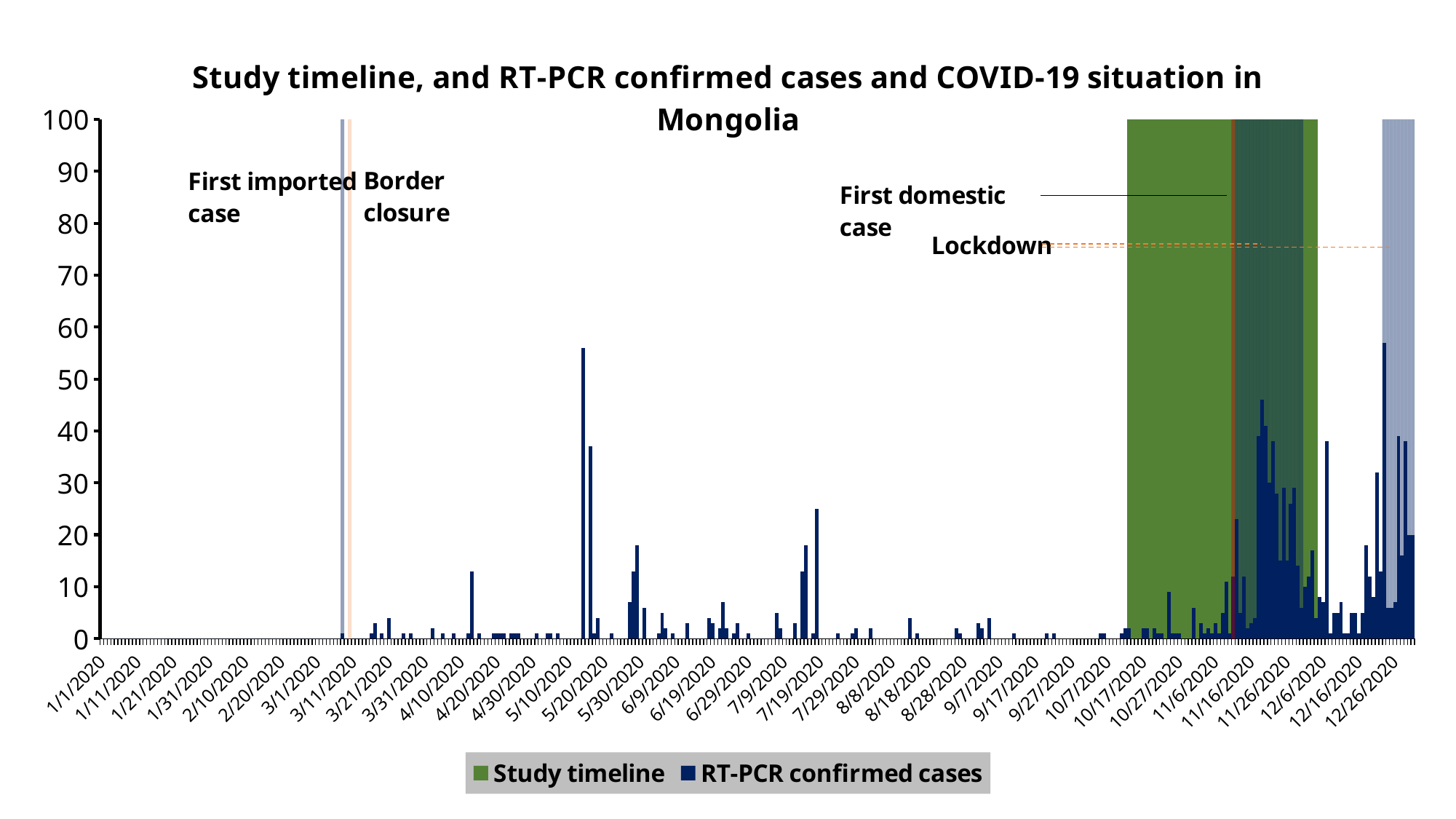

### Chart: Study timeline, and RT-PCR confirmed cases and COVID-19 situation in Mongolia
| Category | | | |
|---|---|---|---|
| 43831 | None | 0.0 | None |
| 43832 | None | 0.0 | None |
| 43833 | None | 0.0 | None |
| 43834 | None | 0.0 | None |
| 43835 | None | 0.0 | None |
| 43836 | None | 0.0 | None |
| 43837 | None | 0.0 | None |
| 43838 | None | 0.0 | None |
| 43839 | None | 0.0 | None |
| 43840 | None | 0.0 | None |
| 43841 | None | 0.0 | None |
| 43842 | None | 0.0 | None |
| 43843 | None | 0.0 | None |
| 43844 | None | 0.0 | None |
| 43845 | None | 0.0 | None |
| 43846 | None | 0.0 | None |
| 43847 | None | 0.0 | None |
| 43848 | None | 0.0 | None |
| 43849 | None | 0.0 | None |
| 43850 | None | 0.0 | None |
| 43851 | None | 0.0 | None |
| 43852 | None | 0.0 | None |
| 43853 | None | 0.0 | None |
| 43854 | None | 0.0 | None |
| 43855 | None | 0.0 | None |
| 43856 | None | 0.0 | None |
| 43857 | None | 0.0 | None |
| 43858 | None | 0.0 | None |
| 43859 | None | 0.0 | None |
| 43860 | None | 0.0 | None |
| 43861 | None | 0.0 | None |
| 43862 | None | 0.0 | None |
| 43863 | None | 0.0 | None |
| 43864 | None | 0.0 | None |
| 43865 | None | 0.0 | None |
| 43866 | None | 0.0 | None |
| 43867 | None | 0.0 | None |
| 43868 | None | 0.0 | None |
| 43869 | None | 0.0 | None |
| 43870 | None | 0.0 | None |
| 43871 | None | 0.0 | None |
| 43872 | None | 0.0 | None |
| 43873 | None | 0.0 | None |
| 43874 | None | 0.0 | None |
| 43875 | None | 0.0 | None |
| 43876 | None | 0.0 | None |
| 43877 | None | 0.0 | None |
| 43878 | None | 0.0 | None |
| 43879 | None | 0.0 | None |
| 43880 | None | 0.0 | None |
| 43881 | None | 0.0 | None |
| 43882 | None | 0.0 | None |
| 43883 | None | 0.0 | None |
| 43884 | None | 0.0 | None |
| 43885 | None | 0.0 | None |
| 43886 | None | 0.0 | None |
| 43887 | None | 0.0 | None |
| 43888 | None | 0.0 | None |
| 43889 | None | 0.0 | None |
| 43890 | None | 0.0 | None |
| 43891 | None | 0.0 | None |
| 43892 | None | 0.0 | None |
| 43893 | None | 0.0 | None |
| 43894 | None | 0.0 | None |
| 43895 | None | 0.0 | None |
| 43896 | None | 0.0 | None |
| 43897 | None | 0.0 | None |
| 43898 | None | 1.0 | 100.0 |
| 43899 | None | 0.0 | None |
| 43900 | None | 0.0 | 100.0 |
| 43901 | None | 0.0 | None |
| 43902 | None | 0.0 | None |
| 43903 | None | 0.0 | None |
| 43904 | None | 0.0 | None |
| 43905 | None | 0.0 | None |
| 43906 | None | 1.0 | None |
| 43907 | None | 3.0 | None |
| 43908 | None | 0.0 | None |
| 43909 | None | 1.0 | None |
| 43910 | None | 0.0 | None |
| 43911 | None | 4.0 | None |
| 43912 | None | 0.0 | None |
| 43913 | None | 0.0 | None |
| 43914 | None | 0.0 | None |
| 43915 | None | 1.0 | None |
| 43916 | None | 0.0 | None |
| 43917 | None | 1.0 | None |
| 43918 | None | 0.0 | None |
| 43919 | None | 0.0 | None |
| 43920 | None | 0.0 | None |
| 43921 | None | 0.0 | None |
| 43922 | None | 0.0 | None |
| 43923 | None | 2.0 | None |
| 43924 | None | 0.0 | None |
| 43925 | None | 0.0 | None |
| 43926 | None | 1.0 | None |
| 43927 | None | 0.0 | None |
| 43928 | None | 0.0 | None |
| 43929 | None | 1.0 | None |
| 43930 | None | 0.0 | None |
| 43931 | None | 0.0 | None |
| 43932 | None | 0.0 | None |
| 43933 | None | 1.0 | None |
| 43934 | None | 13.0 | None |
| 43935 | None | 0.0 | None |
| 43936 | None | 1.0 | None |
| 43937 | None | 0.0 | None |
| 43938 | None | 0.0 | None |
| 43939 | None | 0.0 | None |
| 43940 | None | 1.0 | None |
| 43941 | None | 1.0 | None |
| 43942 | None | 1.0 | None |
| 43943 | None | 1.0 | None |
| 43944 | None | 0.0 | None |
| 43945 | None | 1.0 | None |
| 43946 | None | 1.0 | None |
| 43947 | None | 1.0 | None |
| 43948 | None | 0.0 | None |
| 43949 | None | 0.0 | None |
| 43950 | None | 0.0 | None |
| 43951 | None | 0.0 | None |
| 43952 | None | 1.0 | None |
| 43953 | None | 0.0 | None |
| 43954 | None | 0.0 | None |
| 43955 | None | 1.0 | None |
| 43956 | None | 1.0 | None |
| 43957 | None | 0.0 | None |
| 43958 | None | 1.0 | None |
| 43959 | None | 0.0 | None |
| 43960 | None | 0.0 | None |
| 43961 | None | 0.0 | None |
| 43962 | None | 0.0 | None |
| 43963 | None | 0.0 | None |
| 43964 | None | 0.0 | None |
| 43965 | None | 56.0 | None |
| 43966 | None | 0.0 | None |
| 43967 | None | 37.0 | None |
| 43968 | None | 1.0 | None |
| 43969 | None | 4.0 | None |
| 43970 | None | 0.0 | None |
| 43971 | None | 0.0 | None |
| 43972 | None | 0.0 | None |
| 43973 | None | 1.0 | None |
| 43974 | None | 0.0 | None |
| 43975 | None | 0.0 | None |
| 43976 | None | 0.0 | None |
| 43977 | None | 0.0 | None |
| 43978 | None | 7.0 | None |
| 43979 | None | 13.0 | None |
| 43980 | None | 18.0 | None |
| 43981 | None | 0.0 | None |
| 43982 | None | 6.0 | None |
| 43983 | None | 0.0 | None |
| 43984 | None | 0.0 | None |
| 43985 | None | 0.0 | None |
| 43986 | None | 1.0 | None |
| 43987 | None | 5.0 | None |
| 43988 | None | 2.0 | None |
| 43989 | None | 0.0 | None |
| 43990 | None | 1.0 | None |
| 43991 | None | 0.0 | None |
| 43992 | None | 0.0 | None |
| 43993 | None | 0.0 | None |
| 43994 | None | 3.0 | None |
| 43995 | None | 0.0 | None |
| 43996 | None | 0.0 | None |
| 43997 | None | 0.0 | None |
| 43998 | None | 0.0 | None |
| 43999 | None | 0.0 | None |
| 44000 | None | 4.0 | None |
| 44001 | None | 3.0 | None |
| 44002 | None | 0.0 | None |
| 44003 | None | 2.0 | None |
| 44004 | None | 7.0 | None |
| 44005 | None | 2.0 | None |
| 44006 | None | 0.0 | None |
| 44007 | None | 1.0 | None |
| 44008 | None | 3.0 | None |
| 44009 | None | 0.0 | None |
| 44010 | None | 0.0 | None |
| 44011 | None | 1.0 | None |
| 44012 | None | 0.0 | None |
| 44013 | None | 0.0 | None |
| 44014 | None | 0.0 | None |
| 44015 | None | 0.0 | None |
| 44016 | None | 0.0 | None |
| 44017 | None | 0.0 | None |
| 44018 | None | 0.0 | None |
| 44019 | None | 5.0 | None |
| 44020 | None | 2.0 | None |
| 44021 | None | 0.0 | None |
| 44022 | None | 0.0 | None |
| 44023 | None | None | None |
| 44024 | None | 3.0 | None |
| 44025 | None | None | None |
| 44026 | None | 13.0 | None |
| 44027 | None | 18.0 | None |
| 44028 | None | 0.0 | None |
| 44029 | None | 1.0 | None |
| 44030 | None | 25.0 | None |
| 44031 | None | None | None |
| 44032 | None | None | None |
| 44033 | None | None | None |
| 44034 | None | None | None |
| 44035 | None | None | None |
| 44036 | None | 1.0 | None |
| 44037 | None | None | None |
| 44038 | None | None | None |
| 44039 | None | None | None |
| 44040 | None | 1.0 | None |
| 44041 | None | 2.0 | None |
| 44042 | None | None | None |
| 44043 | None | None | None |
| 44044 | None | None | None |
| 44045 | None | 2.0 | None |
| 44046 | None | None | None |
| 44047 | None | None | None |
| 44048 | None | None | None |
| 44049 | None | None | None |
| 44050 | None | None | None |
| 44051 | None | None | None |
| 44052 | None | None | None |
| 44053 | None | None | None |
| 44054 | None | None | None |
| 44055 | None | None | None |
| 44056 | None | 4.0 | None |
| 44057 | None | None | None |
| 44058 | None | 1.0 | None |
| 44059 | None | None | None |
| 44060 | None | None | None |
| 44061 | None | None | None |
| 44062 | None | None | None |
| 44063 | None | None | None |
| 44064 | None | None | None |
| 44065 | None | None | None |
| 44066 | None | None | None |
| 44067 | None | None | None |
| 44068 | None | None | None |
| 44069 | None | 2.0 | None |
| 44070 | None | 1.0 | None |
| 44071 | None | None | None |
| 44072 | None | None | None |
| 44073 | None | None | None |
| 44074 | None | None | None |
| 44075 | None | 3.0 | None |
| 44076 | None | 2.0 | None |
| 44077 | None | None | None |
| 44078 | None | 4.0 | None |
| 44079 | None | None | None |
| 44080 | None | None | None |
| 44081 | None | None | None |
| 44082 | None | None | None |
| 44083 | None | None | None |
| 44084 | None | None | None |
| 44085 | None | 1.0 | None |
| 44086 | None | None | None |
| 44087 | None | None | None |
| 44088 | None | None | None |
| 44089 | None | None | None |
| 44090 | None | None | None |
| 44091 | None | None | None |
| 44092 | None | None | None |
| 44093 | None | None | None |
| 44094 | None | 1.0 | None |
| 44095 | None | None | None |
| 44096 | None | 1.0 | None |
| 44097 | None | None | None |
| 44098 | None | None | None |
| 44099 | None | None | None |
| 44100 | None | None | None |
| 44101 | None | None | None |
| 44102 | None | None | None |
| 44103 | None | None | None |
| 44104 | None | None | None |
| 44105 | None | None | None |
| 44106 | None | None | None |
| 44107 | None | None | None |
| 44108 | None | None | None |
| 44109 | None | 1.0 | None |
| 44110 | None | 1.0 | None |
| 44111 | None | None | None |
| 44112 | None | None | None |
| 44113 | None | None | None |
| 44114 | None | None | None |
| 44115 | None | 1.0 | None |
| 44116 | None | 2.0 | None |
| 44117 | 100.0 | 2.0 | None |
| 44118 | 100.0 | None | None |
| 44119 | 100.0 | None | None |
| 44120 | 100.0 | None | None |
| 44121 | 100.0 | 2.0 | None |
| 44122 | 100.0 | 2.0 | None |
| 44123 | 100.0 | None | None |
| 44124 | 100.0 | 2.0 | None |
| 44125 | 100.0 | 1.0 | None |
| 44126 | 100.0 | 1.0 | None |
| 44127 | 100.0 | None | None |
| 44128 | 100.0 | 9.0 | None |
| 44129 | 100.0 | 1.0 | None |
| 44130 | 100.0 | 1.0 | None |
| 44131 | 100.0 | 1.0 | None |
| 44132 | 100.0 | None | None |
| 44133 | 100.0 | None | None |
| 44134 | 100.0 | None | None |
| 44135 | 100.0 | 6.0 | None |
| 44136 | 100.0 | None | None |
| 44137 | 100.0 | 3.0 | None |
| 44138 | 100.0 | 1.0 | None |
| 44139 | 100.0 | 2.0 | None |
| 44140 | 100.0 | 1.0 | None |
| 44141 | 100.0 | 3.0 | None |
| 44142 | 100.0 | 1.0 | None |
| 44143 | 100.0 | 5.0 | None |
| 44144 | 100.0 | 11.0 | None |
| 44145 | 100.0 | 1.0 | None |
| 44146 | 100.0 | 12.0 | 100.0 |
| 44147 | 100.0 | 23.0 | 100.0 |
| 44148 | 100.0 | 5.0 | 100.0 |
| 44149 | 100.0 | 12.0 | 100.0 |
| 44150 | 100.0 | 2.0 | 100.0 |
| 44151 | 100.0 | 3.0 | 100.0 |
| 44152 | 100.0 | 4.0 | 100.0 |
| 44153 | 100.0 | 39.0 | 100.0 |
| 44154 | 100.0 | 46.0 | 100.0 |
| 44155 | 100.0 | 41.0 | 100.0 |
| 44156 | 100.0 | 30.0 | 100.0 |
| 44157 | 100.0 | 38.0 | 100.0 |
| 44158 | 100.0 | 28.0 | 100.0 |
| 44159 | 100.0 | 15.0 | 100.0 |
| 44160 | 100.0 | 29.0 | 100.0 |
| 44161 | 100.0 | 15.0 | 100.0 |
| 44162 | 100.0 | 26.0 | 100.0 |
| 44163 | 100.0 | 29.0 | 100.0 |
| 44164 | 100.0 | 14.0 | 100.0 |
| 44165 | 100.0 | 6.0 | 100.0 |
| 44166 | 100.0 | 10.0 | None |
| 44167 | 100.0 | 12.0 | None |
| 44168 | 100.0 | 17.0 | None |
| 44169 | 100.0 | 4.0 | None |
| 44170 | None | 8.0 | None |
| 44171 | None | 7.0 | None |
| 44172 | None | 38.0 | None |
| 44173 | None | 1.0 | None |
| 44174 | None | 5.0 | None |
| 44175 | None | 5.0 | None |
| 44176 | None | 7.0 | None |
| 44177 | None | 1.0 | None |
| 44178 | None | 1.0 | None |
| 44179 | None | 5.0 | None |
| 44180 | None | 5.0 | None |
| 44181 | None | 1.0 | None |
| 44182 | None | 5.0 | None |
| 44183 | None | 18.0 | None |
| 44184 | None | 12.0 | None |
| 44185 | None | 8.0 | None |
| 44186 | None | 32.0 | None |
| 44187 | None | 13.0 | None |
| 44188 | None | 57.0 | 100.0 |
| 44189 | None | 6.0 | 100.0 |
| 44190 | None | 6.0 | 100.0 |
| 44191 | None | 7.0 | 100.0 |
| 44192 | None | 39.0 | 100.0 |
| 44193 | None | 16.0 | 100.0 |
| 44194 | None | 38.0 | 100.0 |
| 44195 | None | 20.0 | 100.0 |
| 44196 | None | 20.0 | 100.0 |
