# Supplementary material for: SARS-CoV-2 seroprevalence in Mongolia: Results from a national population survey
Source: Lancet Reg Health West Pac. 2021 Nov 23;17:100317. doi: 10.1016/j.lanwpc.2021.100317 (PMC8609908; doi:10.1016/j.lanwpc.2021.100317)
Supplement: Supplementary file 3 [file mmc3.docx]

**Statistical Appendix**

**Population Weighting**

We applied a statistical weighting by age, sex, and aimag (province) to account for differences in sampling frequency among these categories. Weights within categories were calculated separately as follows. The proportion of each population segment in the total population (derived from Mongolian Statistical Office 2020 data) was divided by the proportion of the population segment within our survey data. So, for example, to calculate weights by sex, we divided the percentage of males in the total 2020 population by the percentage of males in our survey:

|  | $w_{s}(\mathrm{male})= \frac{p_{m}(total population)}{p_{m}(survey)}$ | (1) |
| --- | --- | --- |

Where $p_{m}$ is the proportion of males. Weights are computed likewise for females, age groups, and aimags. Total weights for each age-sex-aimag group were calculated as the multiple of the weights within each category $w_{t}= w_{a}*w_{s}*w_{aimag}$, and were verified for accuracy via raking. When stratifying by a weighted category, the corresponding weight was removed from the estimate, e.g. when calculating weighted seroprevalence within aimags an age-sex weight was used.

**Adjustment for test kit performance**

Following the analysis in Poustchi et al. 2021 and Bendavid et al. 2021, we adjusted the weighted estimates for performance of the WANTAI SARS-CoV-2 Ab ELISA test using the following formula:

|  | $\pi= \frac{p+s-1}{r+s-1}$ | (2) |
| --- | --- | --- |

Where π is the seroprevalence of SARS-Cov-2 within the general population, p is the seroprevalence within our survey, r is the sensitivity of the WANTAI test, and s is the specificity of the WANTAI test. The WANTAI test’s official sensitivity and specificity are **94.36%** (90.87%-96.81%) and **100%** (98.80%-100%) respectively.

**Bootstrapping**

We applied a bootstrapping method to calculate error on the crude prevalence rate, using the same algorithm as that described in Bendavid et al. 2021. Namely, Bootstrap samples were drawn from both the WANTAI sensitivity data and the Mongolia survey sample and then used to calculate the desired prevalence rates. 10,000 samples were used for the final estimate. Bootstrapping was further used to propagate error through our weighting and test adjustment calculations.

**References**

1. Bendavid, E., Mulaney, B., Sood, N., Shah, S., Bromley-Dulfano, R., Lai, C., ... & Bhattacharya, J. (2021). Covid-19 antibody seroprevalence in santa clara county, california. *International journal of epidemiology*, *50*(2), 410-419.
2. Poustchi, H., Darvishian, M., Mohammadi, Z., Shayanrad, A., Delavari, A., Bahadorimonfared, A., ... & Malekzadeh, R. (2021). SARS-CoV-2 antibody seroprevalence in the general population and high-risk occupational groups across 18 cities in Iran: a population-based cross-sectional study. *The Lancet Infectious Diseases*, *21*(4), 473-481.

**Regional Maps and Tables**


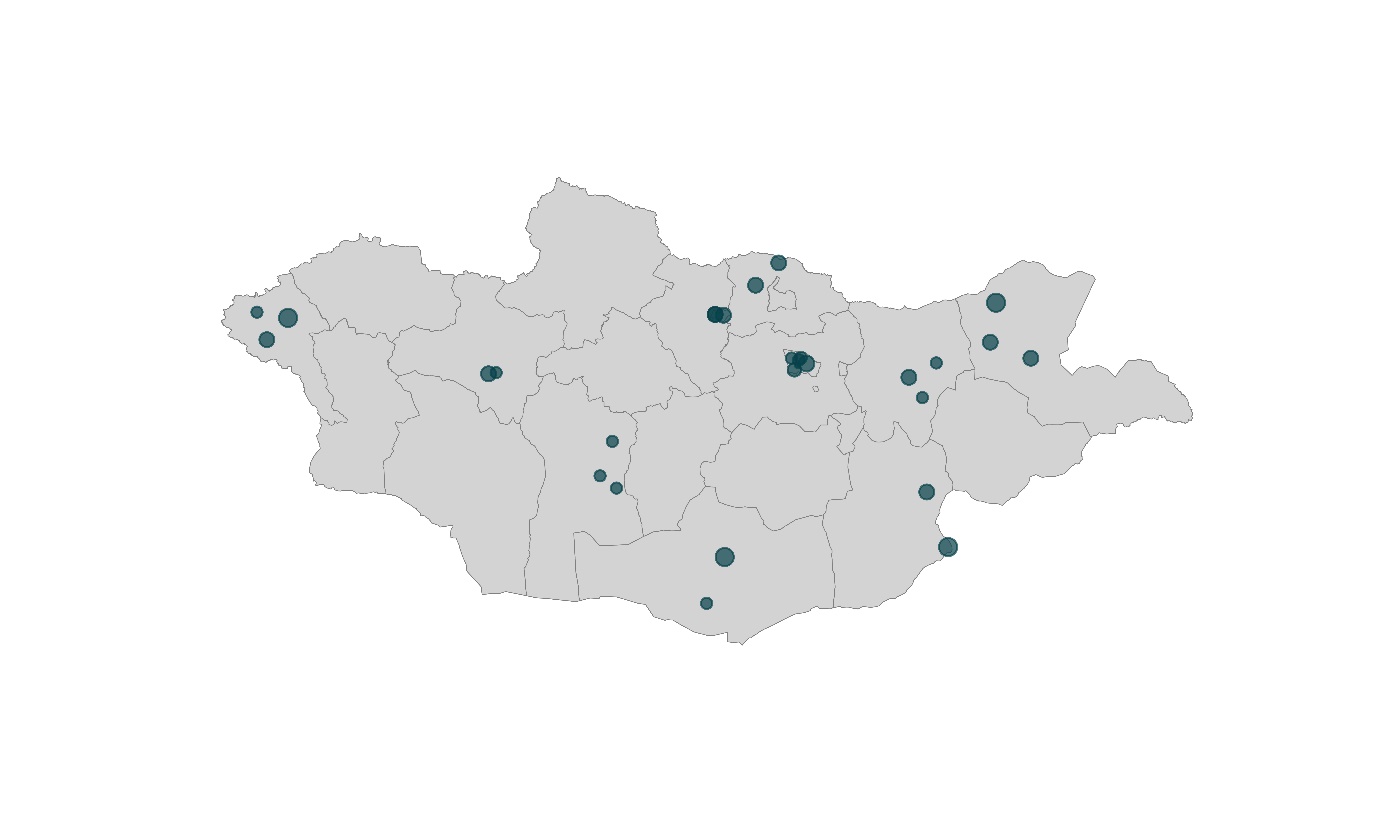


**Figure 1:** Weighted prevalence rate by soum

**Table 1:** Prevalence rate of SARS-CoV-2 antibodies in Mongolia by province

| **Aimag** | **N, total** | **n, seropositive** | **Crude prevalence rate*** | **Weighted prevalence rate** | **Mean of population weights** |
| --- | --- | --- | --- | --- | --- |
| Bayan-Ulgii | 300 | 6 | 2.0 (0.97-3.19) | 1.77 (0.83-2.85) | 0.971400518 |
| Bayankhongor | 300 | 3 | 1.0 (0.32-1.86) | 0.8 (0.26-1.51) | 0.976348979 |
| Dornogovi | 300 | 5 | 1.66 (0.69-2.78) | 1.32 (0.55-2.2) | 0.979235363 |
| Dornod | 300 | 7 | 2.34 (1.21-3.63) | 1.87 (0.97-2.91) | 0.96254792 |
| Zavkhan | 300 | 3 | 1.0 (0.32-1.88) | 0.74 (0.24-1.39) | 1.003561622 |
| Umnugovi | 300 | 4 | 1.33 (0.5-2.33) | 1.5 (0.51-2.64) | 0.959884212 |
| Selenge | 300 | 4 | 1.33 (0.5-2.32) | 1.22 (0.42-2.18) | 0.989541318 |
| Khentii | 300 | 4 | 1.34 (0.5-2.33) | 1.41 (0.49-2.48) | 1.009088481 |
| Orkhon | 300 | 6 | 2.01 (0.97-3.22) | 2.35 (1.09-3.8) | 0.99165493 |
| Ulaanbaatar | 2300 | 30 | 1.3 (0.99-1.64) | 1.31 (0.97-1.66) | 1.02171333 |

(95%CI, Lower and Upper Bound), *- values are not exact seropositive percentage due to bootstrapping

**Table 2:** Prevalence rate of SARS-CoV-2 antibodies in Mongolia by soum and Ulaanbaatar districts

| **Aimag** | **Soum/Duureg** | **N, total** | **n, seropositive** | **Crude prevalence rate*** | **Weighted prevalence rate** | **Mean of population weights** |
| --- | --- | --- | --- | --- | --- | --- |
| Bayankhongor | Bayankhongor | 100 | 1 | 1.16 (0.46-2.62) | 0.93 (0.36-2.09) | 1.012855713 |
| Bayankhongor | Bogd | 100 | 1 | 1.16 (0.46-2.62) | 0.9 (0.35-2.06) | 0.958517579 |
| Bayankhongor | Jinst | 100 | 1 | 1.15 (0.46-2.65) | 0.98 (0.38-2.25) | 0.957673644 |
| Bayan-Ulgii | Ulgii | 100 | 3 | 3.0 (0.97-5.56) | 2.49 (0.79-4.62) | 0.941794846 |
| Bayan-Ulgii | Sagsai | 100 | 2 | 2.02 (0.49-4.17) | 1.56 (0.37-3.22) | 0.988429906 |
| Bayan-Ulgii | Ulaankhus | 100 | 1 | 1.15 (0.46-2.63) | 1.49 (0.59-3.38) | 0.9839768 |
| Dornogovi | Sainshand | 100 | 0 | 0.0 (0.0-0.0) | 0.0 (0.0-0.0) | 0.991984656 |
| Dornogovi | Urgun | 100 | 2 | 2.05 (0.5-4.24) | 1.65 (0.4-3.44) | 0.964027722 |
| Dornogovi | Zamiin-Uud | 100 | 3 | 3.01 (0.96-5.6) | 2.37 (0.75-4.44) | 0.981693712 |
| Dornod | Kherlen | 100 | 2 | 2.04 (0.5-4.19) | 1.55 (0.37-3.2) | 0.965035598 |
| Dornod | Tsagaan-Ovoo | 100 | 2 | 2.03 (0.5-4.17) | 1.5 (0.36-3.1) | 1.014057369 |
| Dornod | Bayandun | 100 | 3 | 3.0 (0.97-5.56) | 2.68 (0.86-5.0) | 0.908550793 |
| Zavkhan | Uliastai | 100 | 1 | 1.16 (0.46-2.67) | 0.85 (0.33-1.96) | 1.01837629 |
| Zavkhan | Aldarkhaan | 100 | 2 | 2.04 (0.5-4.17) | 1.51 (0.36-3.09) | 1.005824555 |
| Zavkhan | Tsagaankhairkhan | 100 | 0 | 0.0 (0.0-0.0) | 0.0 (0.0-0.0) | 0.986484022 |
| Selenge | Sukhbaatar | 100 | 2 | 2.02 (0.49-4.17) | 2.24 (0.43-4.69) | 0.989689107 |
| Selenge | Altanbulag | 100 | 0 | 0.0 (0.0-0.0) | 0.0 (0.0-0.0) | 0.980941245 |
| Selenge | Khushaat | 100 | 2 | 2.05 (0.5-4.17) | 1.48 (0.35-3.01) | 0.997993603 |
| Ulaanbaatar | Bayangol | 400 | 1 | 0.29 (0.12-0.65) | 0.23 (0.09-0.51) | 1.031704065 |
| Ulaanbaatar | Bayanzurkh | 400 | 9 | 2.25 (1.3-3.35) | 2.59 (1.46-3.89) | 1.02585196 |
| Ulaanbaatar | Sukhbaatar | 300 | 4 | 1.33 (0.5-2.34) | 1.22 (0.4-2.2) | 1.029469563 |
| Ulaanbaatar | Songinokhairkhan | 500 | 6 | 1.2 (0.59-1.93) | 1.16 (0.52-1.91) | 1.008374948 |
| Ulaanbaatar | Khan-Uul | 300 | 5 | 1.67 (0.7-2.76) | 1.76 (0.73-2.96) | 1.027643664 |
| Ulaanbaatar | Chingeltei | 400 | 5 | 1.25 (0.51-2.07) | 1.06 (0.44-1.8) | 1.01401699 |
| Khentii | Kherlen | 100 | 2 | 2.05 (0.5-4.17) | 2.17 (0.44-4.47) | 0.985676614 |
| Khentii | Bayankhutag | 100 | 1 | 1.16 (0.46-2.7) | 1.58 (0.62-3.64) | 1.059729883 |
| Khentii | Batnorov | 100 | 1 | 1.17 (0.46-2.67) | 0.86 (0.33-1.97) | 0.981858945 |
| Orkhon | Bayan-Undur, 1 | 100 | 2 | 2.03 (0.49-4.13) | 2.23 (0.42-4.69) | 1.024361066 |
| Orkhon | Jargalant | 100 | 2 | 2.05 (0.49-4.21) | 2.04 (0.48-4.2) | 0.94904399 |
| Orkhon | Bayan-Undur, 3 | 100 | 2 | 2.05 (0.49-4.19) | 2.89 (0.7-5.87) | 1.001559734 |
| Umnugovi | Dalanzadgad | 100 | 3 | 3.0 (0.97-5.53) | 3.79 (1.11-7.12) | 0.92337829 |
| Umnugovi | Khankhongor | 100 | 0 | 0.0 (0.0-0.0) | 0.0 (0.0-0.0) | 0.998559327 |
| Umnugovi | Khurmen | 100 | 1 | 1.17 (0.46-2.65) | 0.99 (0.38-2.25) | 0.957715019 |

(95%CI, Lower and Upper Bound), *- values are not exact seropositive percentage due to bootstrapping


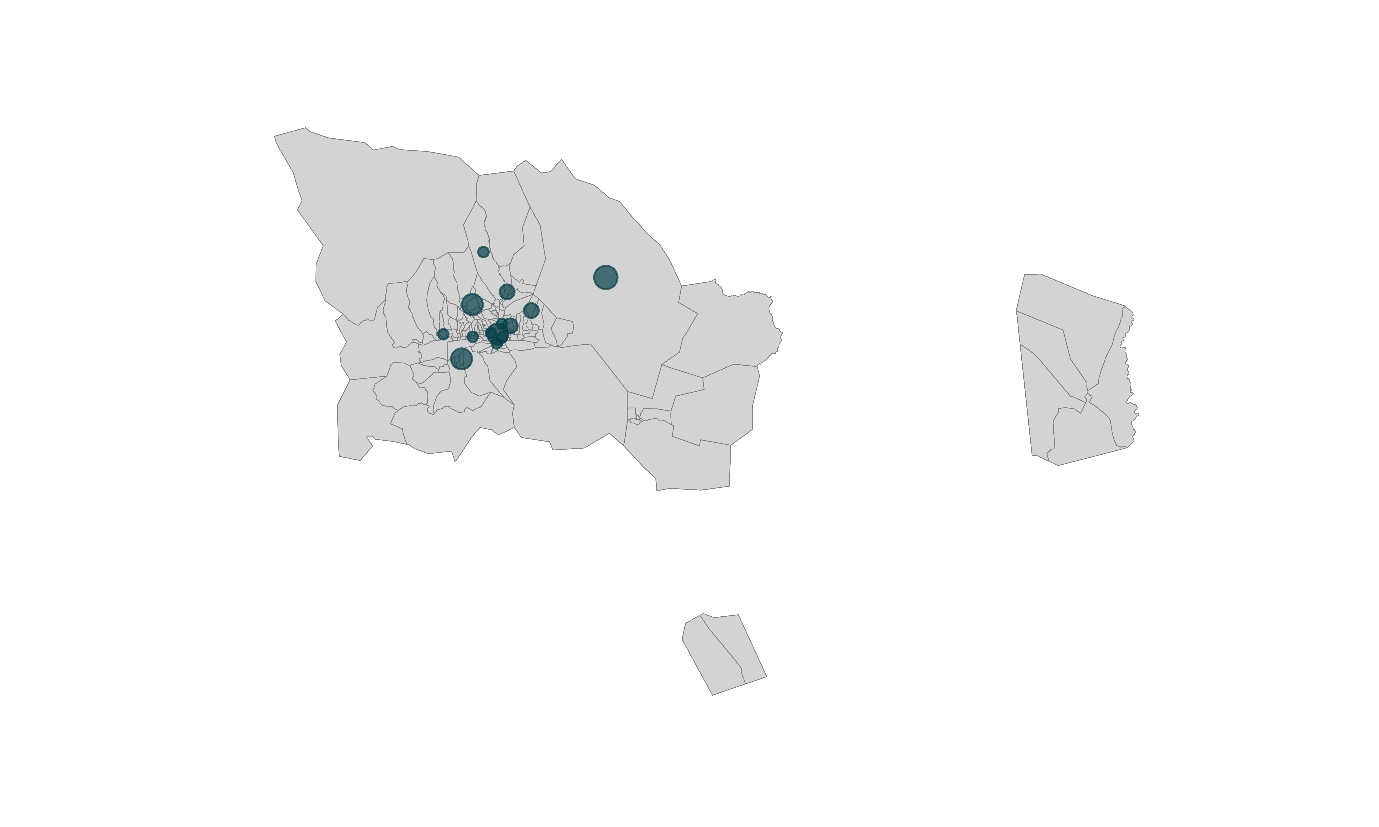


**Figure 2:** Weighted prevalence rate by Ulaanbaatar khoroo

**Table 3:** Prevalence rate of SARS-CoV-2 antibodies in Mongolia by Ulaanbaatar khoroo. Khoroos without SARS-Covid-2 cases are omitted.

| **Duureg** | **Khoroo** | **N, total** | **n, seropositive** | **Crude prevalence rate** | **Weighted prevalence rate** | **Mean of population weights** |
| --- | --- | --- | --- | --- | --- | --- |
| Bayangol | 16 | 100 | 1 | 1.01 (0.23-2.01) | 0.89 (0.2-1.76) | 1.013178483 |
| Bayanzurkh | 2 | 100 | 2 | 2.0 (0.76-3.4) | 2.5 (0.98-4.24) | 1.072874373 |
| Bayanzurkh | 20 | 100 | 5 | 5.0 (3.02-7.14) | 5.27 (3.19-7.55) | 1.022259079 |
| Bayanzurkh | 24 | 100 | 2 | 2.01 (0.75-3.43) | 2.13 (0.79-3.72) | 1.031468578 |
| Sukhbaatar | 1 | 100 | 1 | 1.01 (0.23-2.07) | 0.86 (0.2-1.78) | 1.037461134 |
| Sukhbaatar | 11 | 100 | 1 | 1.01 (0.23-2.03) | 0.83 (0.19-1.66) | 0.99094617 |
| Sukhbaatar | 17 | 100 | 2 | 2.01 (0.74-3.4) | 2.05 (0.78-3.49) | 1.060001385 |
| Songinokhairkhan | 2 | 100 | 1 | 1.01 (0.23-2.04) | 0.83 (0.19-1.66) | 0.985282809 |
| Songinokhairkhan | 10 | 100 | 4 | 4.01 (2.22-5.95) | 4.12 (2.27-6.18) | 1.041926875 |
| Songinokhairkhan | 13 | 100 | 1 | 1.0 (0.23-2.04) | 0.76 (0.17-1.53) | 0.978401341 |
| Khan-Uul | 1 | 100 | 1 | 1.01 (0.23-2.03) | 0.92 (0.21-1.83) | 1.052082531 |
| Khan-Uul | 5 | 100 | 4 | 4.0 (2.26-5.94) | 4.46 (2.46-6.74) | 1.038029355 |
| Chingeltei | 1 | 100 | 4 | 4.0 (2.27-5.98) | 3.22 (1.81-4.84) | 0.970334027 |
| Chingeltei | 19 | 100 | 1 | 1.01 (0.23-2.06) | 1.18 (0.27-2.4) | 1.046880662 |

**Table 4:** 2020 population by weighting categories

|  | **2020 Population** | **2020 percentage of population** |
| --- | --- | --- |
| **Sex** |  |  |
| Female | 1708563 | 0.508873 |
| Male | 1648979 | 0.491127 |
| **Age Group** |  |  |
| 0-4 | 381639 | 0.113666 |
| 05-9 | 388928 | 0.115837 |
| 10-14 | 299512 | 0.089206 |
| 15-19 | 219508 | 0.065378 |
| 20-29 | 501116 | 0.149251 |
| 30-39 | 570446 | 0.169900 |
| 40-49 | 431089 | 0.128394 |
| 50-59 | 315464 | 0.093957 |
| 60-69 | 164521 | 0.049000 |
| 70+ | 85319 | 0.025411 |
| **Provinces** |  |  |
| Bayan-Ulgii | 110597 | 0.046285 |
| Zavkhan | 72763 | 0.030451 |
| Bayankhongor | 88955 | 0.037228 |
| Orkhon | 107765 | 0.045100 |
| Dornogovi | 71207 | 0.029800 |
| Umnugovi | 70371 | 0.029450 |
| Selenge | 109285 | 0.045736 |
| Dornod | 83087 | 0.034772 |
| Khentii | 78172 | 0.032715 |
| Ulaanbaatar | 1597290 | 0.668464 |
