# Supplementary material for: SARS-CoV-2 seroprevalence in Mongolia: Results from a national population survey
Source: Lancet Reg Health West Pac. 2021 Nov 23;17:100317. doi: 10.1016/j.lanwpc.2021.100317 (PMC8609908; doi:10.1016/j.lanwpc.2021.100317)
Supplement: Supplementary file 4 [file mmc4.pdf]

Disclaimer: This translation in Cyrillic and Traditional Mongolian were submitted by the authors and we reproduce it as supplied. It has not been peer reviewed. Our editorial processes have only been applied to the original abstract in English, which should serve as reference for this manuscript.

Монгол улсын хүн амын дунд 2020 оны төгсгөл үед SARS-CoV-2 -ийн эсрэг эсрэгбиеийн тархалт: хүн амд суурилсан үндэсний судалгааны үр дүн

Үндэслэл

Дэлхий дахинд амьсгалын цочмог халтай хам шинж үүсгэгч титэм вирусийн 2-р хэв шинжийн (SARS-CoV-2)-ийн халдвар бүртгэгдэж эхэлсэн үетэй зэрэгцэн Монгол улс хариу арга хэмжээг 2020 оны эхнээс эрт авч хэрэгжүүлснээр 2020 оны 11 сар хүртэл дотоодын халдвар бүртгэгдээгүй. Бид хүн амын дунд SARS-CoV-2 -ийн эсрэг эсрэгбиеийн тархалтыг тодорхойлох үндэсний судалгааг дотоодод халдвар бүртгэгдэх хүртэл хугацаанд судлан тодорхойллоо.

Аргачлал

Судалгаанд 2020 оны 10-аас 12 дугаар сарын хооронд эрүүл мэндийн анхан шатны байгууллагаар дамжуулан хүн амыг насны бүлэгт суурилсан санамсаргүй түүвэрлэлтийн аргаар сонгон хамруулж, асуумж судалгаа болон ийлдэс цуглуулсан. Судалгаанд оролцогсдын ийлдсэнд SARS-CoV-2-ийн эсрэг нийт эсрэгбиеийг тодорхойлж, эерэг тохиолдлыг SARS-CoV-2-ийн эсрэг IgG илрүүлэх хоёр шатлалт, тоон шинжилгээгээр баталгаажуулсан. Жинлэсэн болон шинжилгээнд төвшрүүлсэн эсрэгбиеийн тархалтыг тооцсон. Ийлдэс эерэг тохиолдолтой хамаарах хувьсуурыг тодорхойлоход хи-квадрат, Фишер эксакт сорил болон бусад шинжилгээг ашиглав.

Үр дүн

Судалгаанд нийт 5000 оролцогч хамруулсан ба SARS-CoV-2-ийн эсрэг эсрэг бие (IgG) 72 тохиолдолд илэрсэн. Судалгааны хугацаанд Монгол улсад SARS-CoV-2 -ийн эсрэг эсрэгбиеийн тархалт 1·44% (95%CI, 1·21-1·67) байсан бол хүн амын дундах жинлэсэн тархалт 1·36% (95%CI, 1·11-1·63), шинжилгээнд төвшрүүлсэн тархалт 1·45% (95%CI, 1·11-1·63) байлаа. Хүн амын нас, хүйс, газарзүйн байршил, ажил мэргэжлийн хүчин зүйлс болон ийлдэс тархварзүйн хооронд холбоо ажиглагдаагүй ($p>0·05$). Мөн ийлдэс судлалын шинжилгээний үр дүн ба өмнөх 3 сарын хугацаанд илэрсэн шинж тэмдэг, эмнэлзүйн хамшинжийн хооронд хамаарал ажиглагдаагүй ($p>0·05$).

Хэлцэмж

Хилийн хязгаарлалт тогтоох, сургалтын байгууллагуудыг хаах, амны хаалт зүүх зэрэг нийгмийн эрүүл мэндийн хариу арга хэмжээг эрт хэрэгжүүлсэнтэй холбоотойгоор цартахлын эхний жилд Монгол улсад улсад SARS-CoV-2-ийн эсрэгбиеийн тархалт бага түвшинд байжээ. Бидний судалгаагаар Монгол улсад 2020 оны 11 сар хүртэл хүн амын дунд халдвар томоохон хэмжээгээр тархаагүй байна. Цар тахлын вирусийн эсрэг 2021, 2022 оны вакцинжуулалтын хөтөлбөрийн дараах дархлал тогтоц болон дотоодын халдварын хөдлөлзүйг нарийвчлан тогтоохын тулд ийлдэс судлалын цуврал судалгаа хийх шаардлагатай.

Санхүүжилт

Дэлхийн эрүүл мэндийн байгууллага (ДЭМБ), ХБНГУ-ын Эрүүл мэндийн яам (BMG)-ны КОВИД-19-ын судалгаа хөгжил болон КОВИД-19 Нэгдмэл хариу арга хэмжээний сангийн санхүүжилттэй ДЭМБ-ын ЮНИТИ судалгааны санаачлага

Түлхүүр үгс:

SARS-CoV-2, КОВИД-19, цар тахал, ийлдэс тархварзүйн судалгаа, Монгол улс, ийлдэс тархварзүй, дархлал тогтоц

ᠬᠠᠷᠢᠴᠠᠭᠤᠯᠤᠯ ᠳᠤ ᠲᠣᠬᠢᠷᠠᠭᠤᠯᠤᠭᠰᠠᠨ ᠲᠡᠩᠴᠡᠭᠦᠯᠡᠯ 1.45 (95%CI, 1.11-1.63) ᠪᠠᠢᠨ᠎ᠠ ᠃ ᠡᠮᠡᠭᠲᠡᠢ 1.21-1.67᠃ ᠪᠠᠢᠭᠰᠠᠨ ᠪᠣᠯ ᠡᠷᠡᠭᠲᠡᠢ ᠬᠦᠮᠦᠨ ᠦ ᠬᠠᠷᠢᠴᠠᠩᠭᠤᠢ ᠲᠡᠩᠴᠡᠭᠦᠯᠡᠯ 1.36% (95%CI, 1.11-1.63) ᠂ ᠵᠢᠷᠦᠮᠯᠡᠯ ᠬᠢᠯᠪᠠᠷ ᠲᠠᠢ SARS-CoV-2 ᠶᠢᠨ ᠡᠰᠡᠷᠭᠦ ᠡᠰᠡᠷᠭᠦ ᠪᠡᠶ᠎ᠡ ᠶᠢᠨ ᠲᠠᠯ᠎ᠠ ᠲᠡᠩᠴᠡᠭᠦᠯᠡᠯ 1.44% (95%CI, ᠡᠰᠡᠷᠭᠦ ᠪᠡᠶ᠎ᠡ ᠶᠢᠨ (IgG) ᠶᠢ ᠦᠵᠡᠭᠦᠯᠦᠨ ᠲᠣᠳᠣᠷᠬᠠᠢᠯᠠᠭᠰᠠᠨ ᠰᠢᠯᠭᠠᠯᠲᠠ ᠃ ᠬᠦᠮᠦᠰ ᠦᠨ ᠦ ᠨᠡᠢᠲᠡᠯᠡᠭᠰᠡᠨ ᠳᠤ ᠬᠦᠮᠦᠰ ᠳᠤ ᠨᠢᠭᠡᠨ 1000 ᠬᠦᠮᠦᠨ ᠳᠤᠲᠠᠮ ᠨᠢᠭᠡᠳᠦᠭᠡᠷ ᠦᠶ᠎ᠡ ᠶᠢᠨ SARS-CoV-2 ᠶᠢᠨ ᠡᠰᠡᠷᠭᠦ ᠲᠣᠭ᠎ᠠ ᠶᠢᠨ ᠪᠠᠢᠳᠠᠯ

ᠪᠣᠯᠤᠨ ᠪᠠᠢᠨ᠎ᠠ ᠬᠠᠷᠢᠴᠠᠭᠤᠯᠤᠨ ᠤ ᠳᠦᠩᠨᠡᠯᠲᠡ ᠃

ᠨᠢᠭᠡᠳᠦᠭᠡᠷ ᠨᠡᠢᠲᠡᠯᠡᠭᠰᠡᠨ ᠦ ᠲᠣᠬᠢᠷᠠᠭᠤᠯᠤᠯᠲᠠ ᠳᠤ ᠰᠠᠷ᠎ᠠ - ᠡᠳᠦᠷ ᠂ ᠬᠦᠢᠰᠦ ᠡᠷᠡᠭᠲᠡᠢ ᠬᠦᠮᠦᠨ ᠡᠰᠡᠷᠭᠦ ᠪᠡᠶ᠎ᠡ ᠶᠢᠨ ᠲᠠᠯ᠎ᠠ ᠲᠡᠩᠴᠡᠭᠦᠯᠡᠯ ᠦᠨ ᠲᠣᠳᠣᠷᠬᠠᠢᠯᠠᠯ ᠃ ᠡᠭᠦᠨ ᠢ ᠦᠵᠡᠭᠦᠯᠦᠨ ᠲᠣᠳᠣᠷᠬᠠᠢᠯᠠᠭᠰᠠᠨ ᠲᠤᠰ ᠬᠠᠷᠢᠴᠠᠭᠤᠯᠤᠨ ᠪᠣᠯ ᠪᠦᠬᠦ ᠪᠦᠯᠦᠭ ᠦᠨ ᠳᠤᠮᠳᠠᠳᠤ ᠃ ᠬᠦᠮᠦᠨ ᠪᠦᠯ ᠬᠠᠷᠢᠴᠠᠭᠤᠯᠤᠯ ᠳᠤ ᠲᠣᠬᠢᠷᠠᠭᠤᠯᠤᠭᠰᠠᠨ ᠲᠣᠳᠣᠷᠬᠠᠢᠯᠠᠭᠰᠠᠨ ᠢ SARS-CoV-2 ᠶᠢᠨ ᠡᠰᠡᠷᠭᠦ IgG ᠡᠰᠡᠷᠭᠦᠪᠡᠶ᠎ᠡ ᠨᠢᠭᠡᠨ ᠰᠢᠯᠭᠠᠯᠲᠠ ᠂ ᠲᠦᠷᠦᠯ ᠶᠢᠨ ᠡᠭᠦᠳᠡᠯ ᠳᠤ SARS-CoV-2 ᠶᠢᠨ ᠡᠰᠡᠷᠭᠦ ᠨᠢᠭᠡᠨ ᠡᠰᠡᠷᠭᠦ ᠪᠡᠶ᠎ᠡ ᠶᠢᠨ ᠲᠣᠬᠢᠷᠠᠭᠤᠯᠤᠯᠲᠠ ᠂ ᠦᠵᠡᠭᠦ ᠡᠷᠬᠢᠯᠡᠯᠲᠡ ᠬᠦᠮᠦᠰ ᠦᠨ ᠪᠦᠬᠦ ᠡᠭᠦᠳᠡᠯ ᠳᠤᠮᠳᠠᠳᠤ ᠃ ᠬᠦᠮᠦᠰ ᠦᠨ ᠳᠤ ᠳᠤᠲᠠᠮ ᠬᠦᠮᠦᠰ ᠬᠦᠢᠰᠦ ᠳᠤᠲᠠᠮ ᠲᠣᠬᠢᠷᠠᠭᠤᠯᠤᠭᠰᠠᠨ ᠲᠠ ᠳᠠᠭᠠᠨ ᠢ ᠪᠣᠯ ᠬᠣᠷᠢᠶ᠎ᠠ ᠨᠢᠭᠡᠳᠦᠭᠡᠷ ᠂ ᠳᠠᠭᠤᠨ ᠬᠦᠢᠰᠦ ᠦ ᠪᠦᠬᠦ ᠶᠢ ᠪᠣᠯ ᠲᠡᠭᠦᠨ ᠡᠷᠡᠭᠲᠡᠢ ᠬᠦᠮᠦᠨ ᠦ ᠨᠠᠰᠤᠨ ᠦ ᠪᠦᠯᠦᠭ ᠲᠠᠢ ᠬᠦᠮᠦᠰ ᠳᠤ 2020 ᠣᠨ ᠤ 90-98 ᠬᠤᠪᠢ ᠶᠢᠨ ᠳᠤ ᠨᠢᠭᠡᠳᠦᠭᠡᠷ ᠰᠠᠭᠤᠷᠢ ᠵᠢᠷᠤᠭ ᠲᠠ ᠰᠠᠨᠠᠭᠳᠠᠯ

ᠨᠢᠭᠡᠳᠦᠭᠡᠷ ᠪᠦᠯᠦᠭᠯᠡᠯ ᠪᠠᠢᠳᠠᠯ ᠢᠶᠠᠷ ᠨᠡᠢᠲᠡᠯᠡᠭᠰᠡᠨ ᠳᠤ ᠬᠦᠮᠦᠨ ᠲᠣᠬᠢᠷᠠᠭᠤᠯᠤᠭᠰᠠᠨ ᠢ ᠃
ᠪᠡᠶ᠎ᠡ ᠶᠢᠨ ᠲᠠᠯ᠎ᠠ ᠲᠡᠩᠴᠡᠭᠦᠯᠡᠯ ᠦᠨ ᠲᠣᠬᠢᠷᠠᠭᠤᠯᠤᠯᠲᠠ ᠲᠣᠭᠠᠴᠠᠭᠠᠨ ᠤ ᠬᠦᠮᠦᠰ ᠦᠨ ᠢ ᠲᠣᠬᠢᠷᠠᠭᠤᠯᠤᠨ ᠳᠤ ᠪᠦᠯᠦᠭᠯᠡᠬᠦ ᠲᠣᠭ᠎ᠠ ᠃ ᠪᠠᠢ ᠡᠮᠡᠭᠲᠡᠢ ᠬᠦᠮᠦᠨ ᠲᠠ ᠲᠣᠭᠠᠳᠠ SARS-CoV-2 — ᠶᠢᠨ ᠡᠰᠡᠷᠭᠦ ᠂ ᠡᠰᠡᠷᠭᠦ ᠲᠡᠷᠡᠭᠦ ᠬᠠᠷᠢᠴᠠᠭᠤᠯᠤᠯᠲᠠ ᠲᠠᠢ 2020 ᠣᠨ ᠤ 11 ᠰᠠᠷ᠎ᠠ ᠶᠢᠨ ᠪᠠᠢᠳᠠᠯ ᠶᠢᠨ ᠲᠣᠭᠠᠴᠠᠭᠠᠨ ᠲᠠ ᠨᠢᠭᠡᠳᠦᠭᠡᠷ ᠲᠤᠰ ᠲᠠᠯᠪᠢᠨ ᠵᠢᠷᠦᠮᠯᠡᠯ ᠬᠢᠯᠪᠠᠷ ᠨᠡᠢᠲᠡ ᠰᠠᠨᠠᠭ᠎ᠠ ᠶᠢᠨ ᠴᠠᠭ ᠢᠶᠠᠷ 2020 ᠣᠨ ᠰᠠᠷ᠎ᠠ ᠰᠢᠨ ᠰᠠᠷ᠎ᠠ ᠶᠢᠨ 2 - 6 ᠰᠠᠷ᠎ᠠ ᠬᠦᠷᠲᠡᠯ᠎ᠡ ᠲᠠ (SARS-CoV-2) — ᠶᠢᠨ ᠨᠢᠭᠡᠳᠦᠭᠡᠷ ᠪᠦᠯᠦᠭᠯᠡᠯᠲᠡ ᠡᠭᠦᠳᠡᠯ ᠲᠣᠭ᠎ᠠ ᠲᠡᠭᠦᠨ ᠲᠡᠭᠡᠷᠡ ᠳᠤ ᠡᠭᠦᠳᠡᠭᠰᠡᠨ ᠶᠢᠨ ᠰᠠᠭᠤᠳᠠᠯ ᠨᠢᠭᠡᠳᠦᠭᠡᠷ ᠨᠢᠭᠡ ᠬᠦᠮᠦᠨ ᠡᠷᠬᠢᠯᠡᠯᠲᠡ ᠲᠡᠭᠦᠨ ᠴᠠᠭ ᠢ ᠲᠣᠭᠠᠴᠠᠭᠠᠯᠲᠠ

ᠬᠦᠮᠦᠰ ᠦᠨ ᠲᠣᠭᠠᠴᠠᠭᠠᠯᠲᠠ ᠶᠢᠨ ᠬᠦᠮᠦᠰ ᠦᠨ ᠤ ᠲᠣᠭ᠎ᠠ ᠶᠢᠨ ᠪᠠᠢᠳᠠᠯ
(SARS-CoV-2) ᠲᠠ ᠡᠰᠡᠷᠭᠦ ᠂ ᠡᠰᠡᠷᠭᠦ ᠪᠡᠶ᠎ᠡ ᠶᠢᠨ ᠲᠠᠯ᠎ᠠ ᠲᠡᠩᠴᠡᠭᠦᠯᠡᠯ : ᠡᠮᠡᠭᠲᠡᠢ ᠬᠦᠮᠦᠨ ᠳᠤ
ᠵᠢᠷᠦᠮᠯᠡᠯ ᠬᠢᠯᠪᠠᠷ ᠶᠢᠨ ᠡᠮᠡᠭᠲᠡᠢ ᠬᠦᠮᠦᠨ ᠲᠠ ᠲᠣᠭᠠᠳᠠ 2020 ᠣᠨ ᠤ ᠲᠠᠪᠤᠳᠤᠭᠠᠷ ᠲᠣᠭ᠎ᠠ ᠶᠢᠨ ᠳᠤ

[illegible]
[illegible]
(p>0.05) :: [illegible]
[illegible]
[illegible] (p>0.05) ::

[illegible]
[illegible]
[illegible]
[illegible]
[illegible] SARS-CoV-2 [illegible]
[illegible]
[illegible]
[illegible]
[illegible]
[illegible] ::

[illegible]
[illegible]
[illegible] (BMG) [illegible]
[illegible]
[illegible]

[illegible] : [illegible]
[illegible]
